# Extracellular Matrix Stiffness Regulates Cancer Stemness in Uveal Melanoma via the PIEZO1–DOT1L Axis

**DOI:** 10.1167/iovs.66.15.57

**Published:** 2025-12-18

**Authors:** Yu Zhang, Jinfeng Cao, Shuyang Zhang, Songtao Wang, Jinrong Cui, Jinsong Zhao

**Affiliations:** 1 Department of Ophthalmology, The Second Hospital of Jilin University, Changchun, China

**Keywords:** uveal melanoma, extracellular matrix stiffness, cancer stemness, PIEZO1, DOT1L

## Abstract

**Purpose:**

Cancer stemness drives aggressive behavior and treatment resistance in uveal melanoma (UM). This study aimed to investigate how mechanical signals from the extracellular matrix (ECM) regulated UM stemness through the piezo-type mechanosensitive ion channel component 1 (PIEZO1)–disruptor of telomeric silencing 1-like (DOT1L) signaling axis.

**Methods:**

PIEZO1 expression was assessed using immunofluorescence in human UM and adjacent normal tissues. Polyacrylamide hydrogel models with tunable stiffness were used to simulate the biomechanical microenvironment in vitro. Stemness was assessed by analyzing colony formation, tumorsphere assays, apoptosis resistance, and expression of the stemness markers NANOG and SOX2. In vivo, ECM stiffness was reduced to examine its effects on UM progression and stemness. The roles of PIEZO1 and DOT1L in ECM stiffness-mediated regulation of stemness were examined both via short-hairpin RNA (shRNA), lentiviral overexpression, and a PIEZO1 agonist.

**Results:**

PIEZO1 was upregulated in UM tissues. In vitro, increased ECM stiffness enhanced UM stemness through PIEZO1. Functioning as a mechanosensor, PIEZO1 promoted DOT1L expression, which consequently upregulated the stemness markers. In vivo, reduced ECM stiffness suppressed tumor growth and downregulated the PIEZO1–DOT1L axis and stemness markers. Inhibition of PIEZO1 or DOT1L diminished stemness properties and tumor growth both in vitro and in vivo.

**Conclusions:**

The PIEZO1–DOT1L axis mediated ECM stiffness–driven stemness and tumor progression in UM. Targeting this mechanotransduction pathway by modulating ECM stiffness or its downstream effectors may provide a novel therapeutic strategy for UM.

Uveal melanoma (UM) is the predominant primary intraocular malignancy in adult populations.^1^^,^^2^ It is highly aggressive and chemoresistant, posing a serious threat to patients’ survival.^3^^–^^5^ Cancer stem cells (CSCs) play a crucial role in UM progression and treatment resistance due to their intrinsic properties of self-renewal, high tumorigenicity, and drug resistance.^6^^–^^9^ Their differentiation heterogeneity contributes to intratumoral complexity,^7^^,^^10^ and their phenotypic plasticity enables dedifferentiation of terminally differentiated tumor cells, allowing them to reacquire stem-like properties under certain conditions.^11^^–^^13^ These features collectively enhance tumor adaptability and drive resistance to therapy.^6^^,^^7^^,^^14^^,^^15^

Notably, the mechanical characteristics of the tumor microenvironment (TME) have emerged as significant regulatory factors influencing CSC plasticity.^12^^,^^16^^–^^18^ During UM progression, the abnormal deposition and cross-linking of extracellular matrix (ECM) components, including collagen, result in significantly increased ECM stiffness.^14^^,^^19^^–^^21^ ECM stiffness, which is a defining mechanical feature in TME, regulates stemness characteristics in various carcinomas, including breast, gastric, and liver cancers, by activating mechanotransduction pathways such as Yes-associated protein/transcriptional coactivator with PDZ-binding motif.^11^^,^^12^^,^^18^ However, the role and potential mechanisms of ECM stiffness in regulating UM stemness remain incompletely understood.

Piezo-type mechanosensitive ion channel component 1 (PIEZO1) is a core mechanosensor that detects ECM stiffness. It regulates gene expression and cellular functions by converting mechanical stimuli into intracellular biochemical signals.^22^^–^^25^ Aykut et al.^26^ discovered that myeloid cells upregulate histone deacetylase 2 upon sensing mechanical stress through PIEZO1, which suppresses the retinoblastoma (Rb1) protein expression, thereby promoting tumor development. Their study revealed that PIEZO1 promotes malignant tumor progression via a mechanotransduction-mediated histone modification signaling axis. PIEZO1 is abnormally overexpressed in multiple tumor types and regulates stemness characteristics to influence tumor progression.^23^^,^^25^ However, the expression patterns and biological functions of PIEZO1 in UM remain unclear.

Epigenetic alterations, particularly histone modifications, are key mechanisms that regulate the phenotypic plasticity of CSCs.^27^^,^^28^ For example, disruptor of telomeric silencing 1-like (DOT1L) promotes tumor progression in gliomas by mediating histone H3K79 methylation (H3K79me), which activates the stemness markers including NANOG and SOX2.^29^ Studies on head and neck cancer have shown that hyaluronic acid (HA), a key component of the ECM, induces DOT1L activation, which consequently promotes tumor cell stemness.^30^ DOT1L is abnormally overexpressed in UM and has been identified as a critical epigenetic driver of UM tumorigenesis and progression.^31^ However, whether ECM stiffness modulates DOT1L activity in UM, thereby affecting cancer stemness, is yet to be elucidated. Considering these key scientific questions, this study aimed to uncover the mechanistic relationship between ECM biomechanics and UM stemness.

## Methods

### Study Design and Ethics

Human UM tissues for immunofluorescence were obtained with informed consent under the approval of the Ethics Committee of The Second Hospital of Jilin University (Approval No. 2025124). Specimens were collected from six treatment-naïve patients (three males and three females) undergoing primary enucleation. The study included only histopathologically confirmed primary UM cases with paired adjacent normal tissues and excluded cases with recurrence, poor tissue quality, or comorbid ocular diseases that could affect ECM structure. All animal procedures complied with the ARVO Statement for the Use of Animals in Ophthalmic and Vision Research and were approved by the Animal Ethics Committee of Changchun Weishi Testing Technology Service Co., Ltd. (Approval No. 20240309-01).

Authenticated UM cell lines and healthy BALB/c nude mice without systemic abnormalities were used as inclusion criteria. To ensure rigor and reproducibility, key experiments—including tumorsphere and colony formation assays, western blotting, quantitative real-time polymerase chain reaction (qRT-PCR), and xenograft studies—were performed in at least three independent experiments. Mice were randomly assigned to treatment groups, and investigators performing quantitative measurements (tumorsphere areas, colony numbers, tumor volumes) were blinded to group allocation.

### Cell Culture

The cell line 92.1^32^ was kindly provided by Martine J. Jager, MD, PhD (Department of Ophthalmology, Leiden University Medical Center, Leiden, Netherlands). The cell line Mel270 was kindly provided by Renbing Jia, MD, PhD, and Yongyun Li, MD (Department of Ophthalmology, Shanghai Ninth People's Hospital, Shanghai Jiao Tong University School of Medicine, Shanghai, China). Both cell lines were cultured in RPMI 1640 Medium supplemented with 10% fetal bovine serum and 1% penicillin–streptomycin.

### Construction of Stabilized Cell Lines

The short-hairpin RNA (shRNA) expression plasmids targeting *PIEZO1* (sh*PIEZO1*) and *DOT1L* (sh*DOT1L*) and the *DOT1L* overexpression plasmid (OE-*DOT1L*) were designed by JTS Scientific (Wuhan, China). Supplementary Table S1 presents the detailed sequences. After infection of cells by lentivirus, stable transfected cell lines were obtained by screening using 2-µg/mL puromycin.

### Preparation and Mechanical Characterization of Polyacrylamide Hydrogels

Polyacrylamide hydrogels (PAAGs) with tunable stiffness were prepared by adjusting the ratio of acrylamide (40%) to *N*,*N*′-methylenebisacrylamide (2%)^33^ (detailed formulations are provided in Supplementary Table S2). The prepolymer solution was sterilized through a 0.22-µm filter. Polymerization was initiated by adding 10% ammonium persulfate and *N*,*N*,*N*′,*N*′-tetramethylethylenediamine (TEMED), after which the mixture was immediately dispensed onto silanized glass slides, covered with coverslips, and allowed to polymerize for 20 minutes at room temperature. To enable cell adhesion, the polymerized gels were activated with Sulfo-SANPAH under ultraviolet light (365 nm, 250 W) for 30 minutes, rinsed with PBS, and subsequently coated with type I collagen (10 µg/mL; GlpBio, Montclair, CA, USA) overnight at 4°C. The mechanical properties of the PAAGs were quantified using an indentation testing device (Supplementary Figs. S1B–S1D).^34^ The shear modulus (*G*) was calculated according to Sneddon's elastic contact model, and the Young's modulus (*E*) was derived using Hooke's law.^35^ Based on these measurements, hydrogel systems spanning Young's moduli from 1 to 39 kPa were generated (Supplementary Table S2).

### Colony Formation Assay

After culturing the cells on PAAG substrates with different stiffnesses, 2000 cells/well were inoculated into six-well plates. Cells were stained with crystal violet (Solarbio, Beijing, China) for visualization and clonogenic capacity quantification following standardized culture.

### Tumorsphere Formation Assay

Ninety-six plates were pretreated with 1.5% agarose (Sevenbio, Beijing, China).^36^ Cells were cultured on PAAG substrates of different stiffnesses and subsequently seeded into 96-well plates at a density of 2000 cells/well. The tumorsphere area was analyzed using ImageJ (National Institutes of Health, Bethesda, MD, USA).

### Cytotoxicity Assay

PAAGs of different stiffnesses were immersed in a complete medium for 48 hours to obtain hydrogel extracts. Cells were treated with complete medium and hydrogel extracts of different stiffnesses. Cell activity was assessed using the Cell Counting Kit-8 (CCK-8) reagent (Sevenbio) at 24, 48, and 72 hours of treatment.

### Apoptosis Analysis

Cells were inoculated with PAAG substrates of different stiffnesses and cultured for 72 hours. The sorafenib (MedChemExpress, Monmouth Junction, NJ, USA)^12^ and dimethyl sulfoxide (DMSO) control treatments were administered for 24 hours. Apoptosis was assessed via flow cytometry utilizing an Annexin V-PE/7-AAD dual-staining detection kit (Multi Sciences, Hangzhou, China).

### Quantitative Real-Time Polymerase Chain Reaction

Total RNA was isolated using the Total RNA Isolation Kit (Vazyme, Nanjing, China). cDNA synthesis was performed on purified RNA using HiScript III RT SuperMix for qPCR (Vazyme), followed by amplification with ChamQ Universal SYBR qPCR Master Mix (Vazyme). Supplementary Table S1 presents the primer sequences.

### Western Blotting Analysis

Cellular and tissue proteins were separated through sodium dodecyl sulfate–polyacrylamide gel electrophoresis and transferred to polyvinylidene fluoride membranes (MilliporeSigma, Billerica, MA, USA). The membranes were blocked with 5% non-fat milk for 1 hour, followed by 16 hours of incubation at 4°C with primary antibodies (Supplementary Table S3). They were analyzed using horseradish peroxidase–conjugated secondary antibodies, and chemiluminescent signals were captured using an enhanced chemiluminescence substrate.

### Immunofluorescence

Cells were fixed in 4% paraformaldehyde, and tumor tissues were paraffin embedded for immunofluorescence detection of target proteins. Supplementary Table S3 presents the corresponding primary antibodies.

### Calcium Ion Probe Staining Assay

Cells were cultured in 24-well plates, treated with 10-µM Yoda1 (MedChemExpress), and DMSO. Calcium imaging was performed using Rhod-2 AM (MedChemExpress), observing the fluorescence signal.

### Animal Experiments

A subcutaneous xenograft model was created by injecting Mel270 cell suspension into BALB/c nude mice. In the ECM-softened group, β-aminopropionitrile (BAPN; 100 mg/kg/d, intraperitoneal injection)^33^^,^^37^ was administered for 4 weeks. A subcutaneous xenograft tumor model was created using Mel270 cells transfected with sh*PIEZO1*/sh*DOT1L* in the gene intervention group. Tumor volume (*V* = *length* × *width*^2^/2) was measured weekly. The animals were euthanized 36 days post-inoculation, and the tumors were excised and weighed.

### Bioinformatic Survival Analysis

Survival outcomes were evaluated by performing Kaplan–Meier and univariate Cox regression analyses in RStudio (R Foundation for Statistical Computing, Vienna, Austria) based on transcriptomic data from the TCGA uveal melanoma cohort (https://portal.gdc.cancer.gov/).

### Statistical Analysis

All statistical analyses were conducted using the Prism 9.0 (GraphPad, Boston, MA, USA). Continuous variables are expressed as arithmetic mean ± standard deviation (SD). Intergroup comparisons employed Student's *t*-tests (two-group) or analysis of variance variants (multi-group). Statistical significance was set at *P* < 0.05.

## Results

### ECM Stiffness Promotes UM Stemness Characteristics and Drug Resistance In Vitro

To investigate how ECM stiffness influences UM cell behavior, we first established PAAG substrates with defined mechanical properties (Supplementary Fig. S1A).^33^ The ECM stiffness of a normal choroid is reportedly 5.4 kPa,^38^ but the average ECM stiffness of tumors is 20 kPa.^39^ Therefore, we selected PAAGs with stiffness values of 5 kPa (soft substrate) and 21 kPa (stiff substrate) to mimic the UM cellular niche. To assess the biocompatibility of PAAGs, 92.1 and Mel270 cells were cultured in PAAG extracts. CCK-8 analyses revealed no significant differences in cell viability compared with standard culture conditions (Supplementary Fig. S1E), indicating that soluble leachates from the hydrogels exerted no cytotoxic effects.

**Figure 1.**
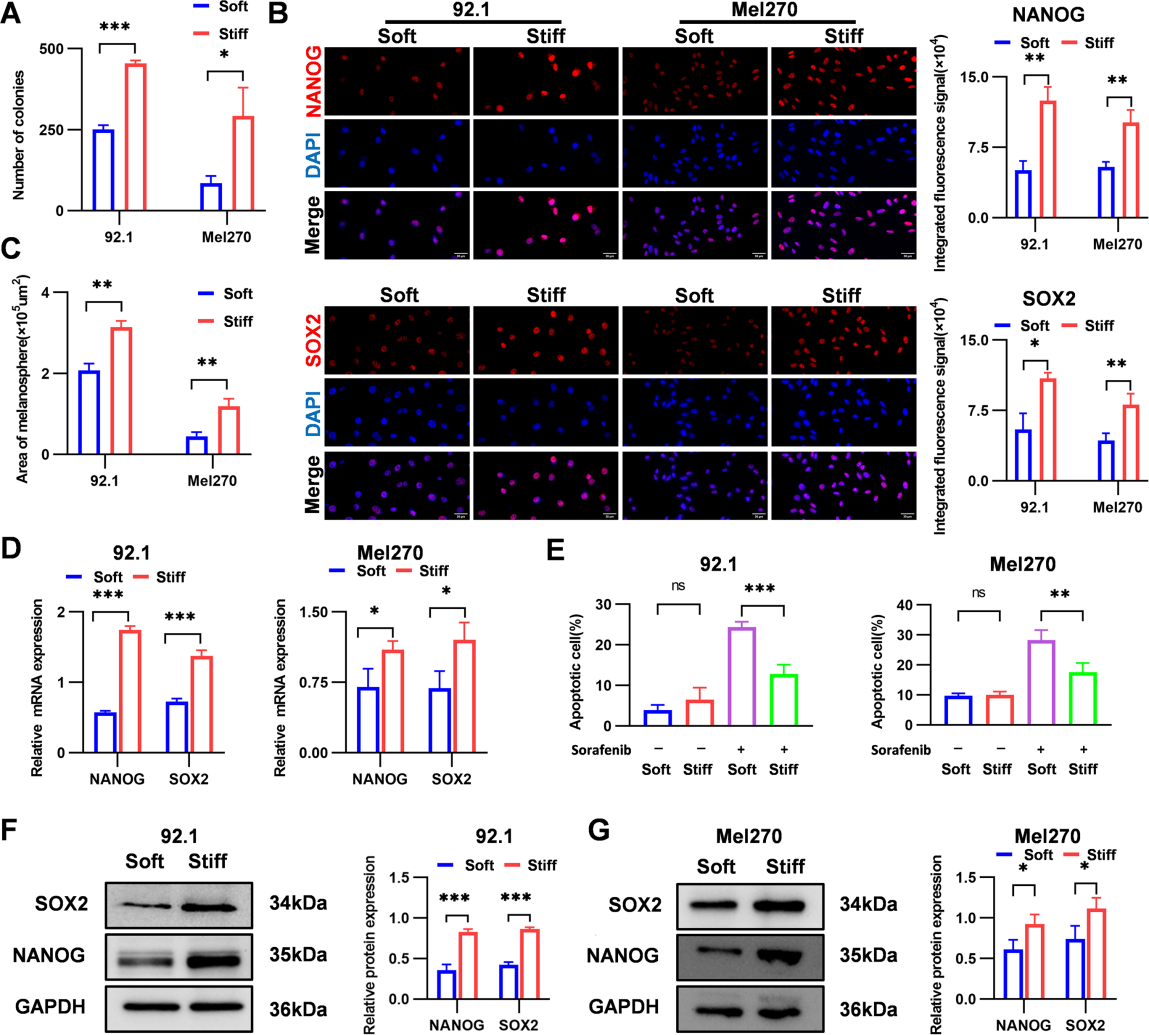
ECM stiffness regulates the stemness characteristics of UM cells in vitro. (**A**) Quantification of colony-forming ability of 92.1 and Mel270 cells cultured on soft and stiff substrates. (**B**) Immunofluorescence images of NANOG and SOX2 proteins (*red*) and DAPI (*blue*) in 92.1 and Mel270 cells cultured on soft and stiff substrates. *Scale bar*: 30 µm. (**C**) Quantification of tumorsphere area under soft- and stiff-substrate conditions. (**D**) The qRT-PCR analysis of *NANOG* and *SOX2* mRNA expression levels in 92.1 and Mel270 cells cultured on soft or stiff substrates. (**E**) Apoptosis rates of 92.1 and Mel270 cells cultured on soft or stiff substrates and treated with sorafenib (10 µM) or DMSO (control) for 24 hours. Apoptosis was assessed by flow cytometry. (**F**, **G**) Western blotting analysis of NANOG and SOX2 protein expression in 92.1 cells (**F**) and Mel270 cells (**G**) cultured on soft and stiff substrates, with GAPDH as a loading control. Data are presented as mean ± SD (*n* = 3). *P < 0.05, **P < 0.01, ***P < 0.001; ns, not significant (*P* > 0.05).

To evaluate the effects of ECM stiffness on UM stemness, 92.1 and Mel270 cells were cultured on soft and stiff PAAG substrates. Stiff substrates markedly enhanced self-renewal capacity in both UM cell lines, with colony formation increasing by 1.8-fold in 92.1 cells and 3.4-fold in Mel270 cells (Fig. 1A; Supplementary Fig. S2A). Tumorsphere area showed a similar trend, increasing by 1.5-fold in 92.1 cells and 2.7-fold in Mel270 cells relative to soft substrates (Fig. 1C; Supplementary Fig. S2B). Next, we investigated whether ECM stiffness influences the sensitivity of UM cells to sorafenib, given the known association between cancer stemness and therapeutic resistance. Flow cytometry showed that the apoptosis rates of 92.1 and Mel270 cells on stiff substrates in the sorafenib-treated group were 12% and 17%, respectively, significantly lower than those on soft substrates (24% and 28%, respectively) (Fig. 1E; Supplementary Fig. S2C). No significant differences were observed in the DMSO control groups (Fig. 1E; Supplementary Fig. S2C), indicating that elevated ECM stiffness does not affect baseline apoptosis but specifically reduces sorafenib-induced apoptosis. At the molecular level, stiff substrates significantly upregulated stemness markers. *NANOG* mRNA increased by 3.0-fold in 92.1 cells and 2.0-fold in Mel270 cells, whereas *SOX2* mRNA increased by 1.6-fold and 1.8-fold, respectively (Fig. 1D). Western blotting showed that stiff substrates significantly elevated the expression of NANOG (2.3-fold in 92.1 cells and 1.5-fold in Mel270 cells) and SOX2 (2.0-fold in 92.1 cells and 1.5-fold in Mel270 cells) (Figs. 1F, 1G). Immunofluorescence also demonstrated increased NANOG and SOX2 expression under stiff substrates (Fig. 1B). Collectively, these results demonstrate that elevated ECM stiffness enhances stem-like properties and confers resistance to targeted therapy in UM cells, highlighting the critical role of biomechanical cues in modulating tumor cell plasticity.

### ECM Softening Suppresses UM Stemness and Tumor Progression In Vivo

**Figure 2.**
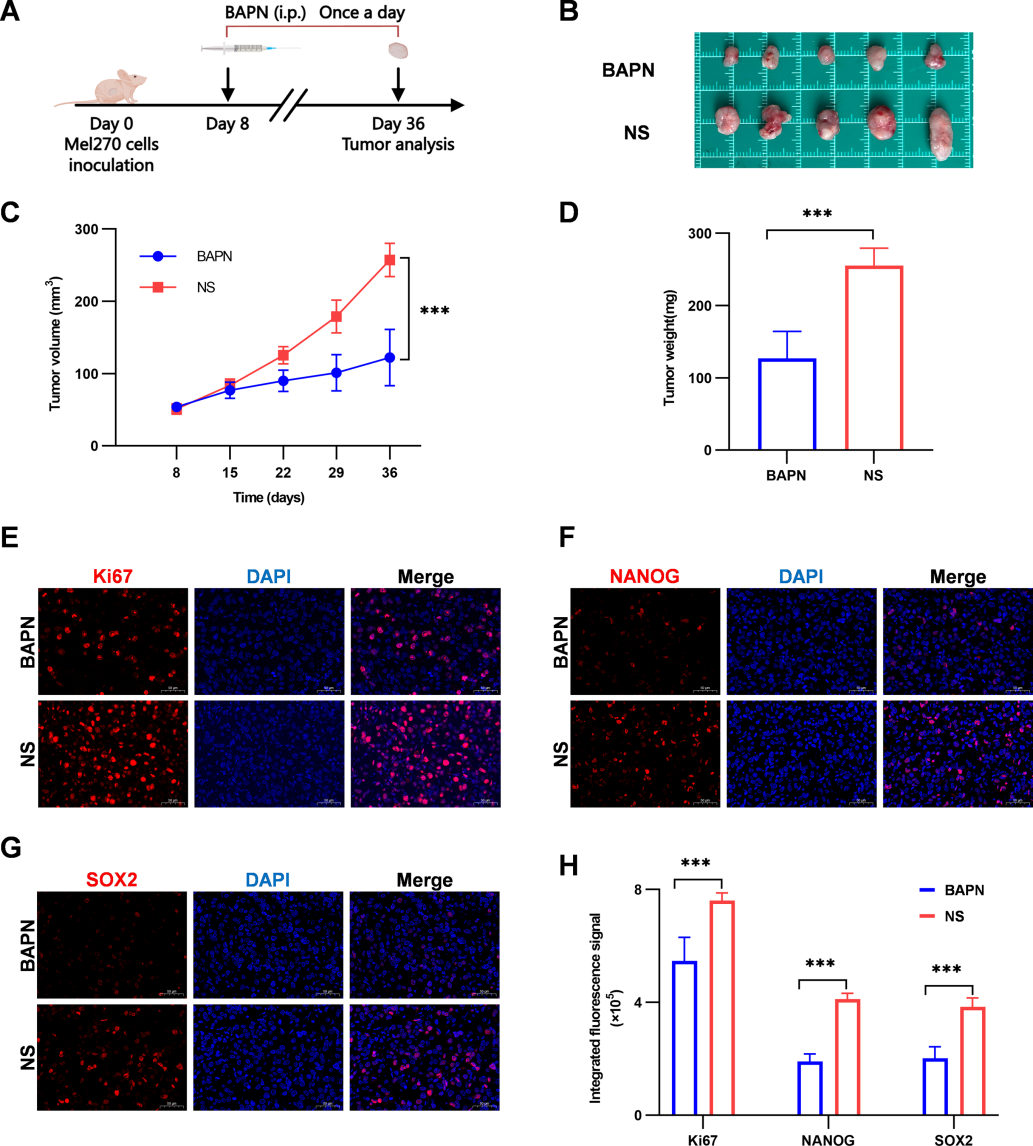
ECM stiffness regulates the stemness of UM cells in vivo. (**A**) Schematic of the experimental design for BAPN treatment in a nude mice subcutaneous tumor model. (**B**) Images of subcutaneous tumors from the BAPN-treated and NS control groups. (**C**) Tumor growth curves for the BAPN-treated and NS control groups. (**D**) Tumor weights in BAPN-treated and NS control groups. (**E**–**G**) Immunofluorescence images of Ki67 (**E**), NANOG (**F**), and SOX2 (**G**) protein expression (*red*) and DAPI (*blue*) in tumor tissues from the BAPN-treated and NS control groups. *Scale* *bar*: 50 µm. (**H**) Quantification of Ki-67-, SOX2-, and NANOG-positive signals corresponding to panels **E** to **G**. Data are presented as mean ± SD (*n* = 5). ***P < 0.001.

To explore whether ECM stiffness promotes UM stemness in vivo, we constructed a subcutaneous xenograft model using Mel270 cells and modulated ECM stiffness using a targeted ECM remodeling strategy. The collagen cross-linking inhibitor BAPN was administered to create a low-stiffness microenvironment,^33^^,^^37^^,^^40^ and the normal saline (NS) group served as the control (Fig. 2A). BAPN treatment did not significantly affect the body weight of the mice (Supplementary Fig. S3A), suggesting the absence of overt systemic toxicity under the current experimental conditions. Masson's trichrome staining showed that collagen fiber density and alignment were reduced by approximately 52% in the BAPN-treated tumors (Supplementary Fig. S4), confirming successful ECM remodeling. ECM softening markedly inhibited tumor growth, resulting in a 53% reduction in volume and a corresponding 50% decrease in final tumor weight by day 36 (Figs. 2B–D). Consistently, Ki67 immunostaining revealed fewer proliferating cells in the BAPN group (Figs. 2E, 2H). Immunofluorescence analysis revealed a 53% decrease in NANOG and a 47% decrease in SOX2 protein expression in BAPN-treated tumors (Figs. 2F–H). Importantly, BAPN treatment did not affect the expression of melanocytic lineage marker Melan-A (Supplementary Fig. S5A), suggesting that ECM softening did not alter tumor cell identity. These in vivo results, combined with our in vitro findings, support the conclusion that ECM stiffness promotes stemness and tumor progression in UM.

### ECM Stiffness Regulates UM Stemness via PIEZO1

**Figure 3.**
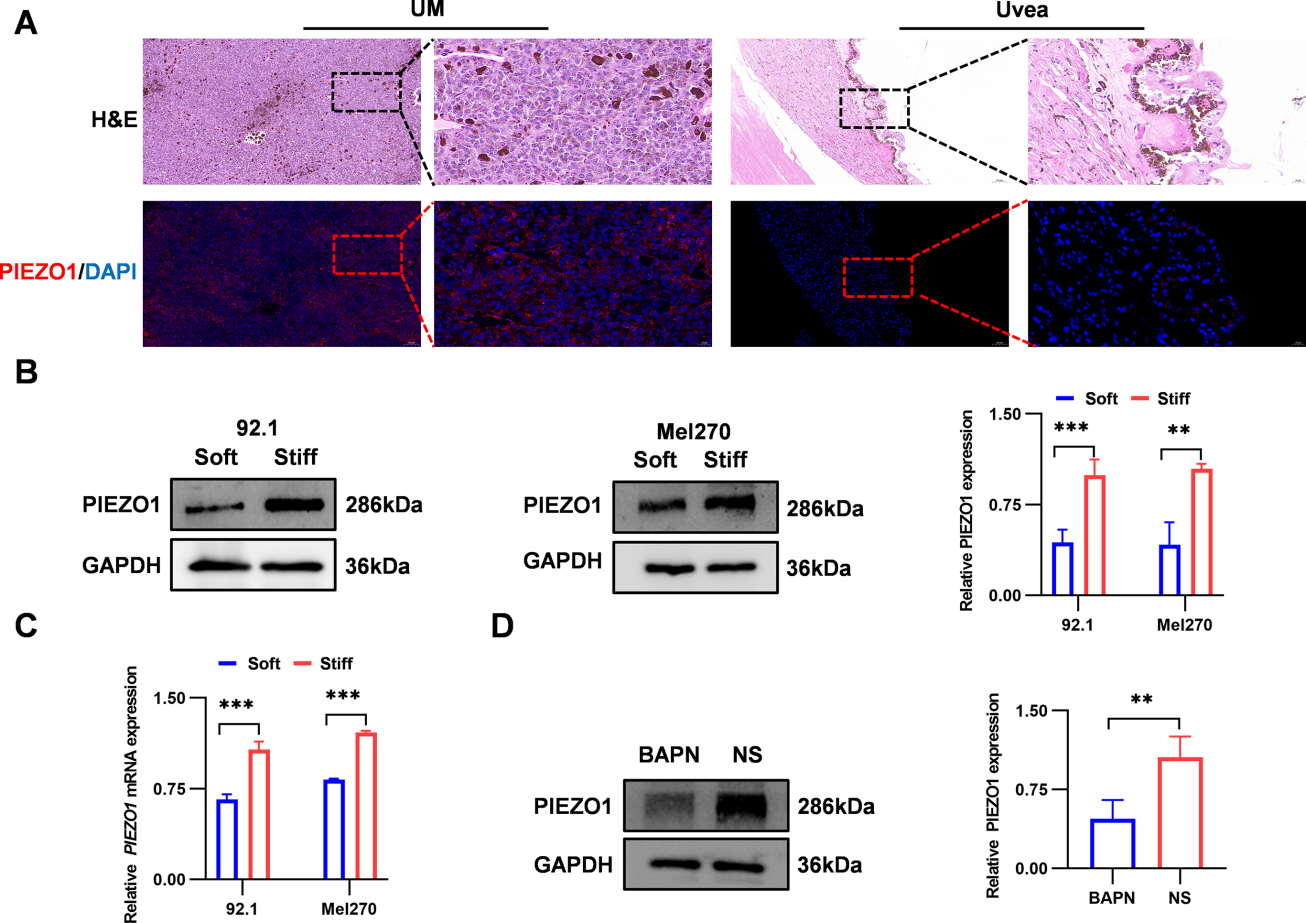
ECM stiffness regulates PIEZO1 expression in vitro and in vivo. (**A**) Hematoxylin and eosin staining and immunofluorescence images of the PIEZO1 protein (*green*) and DAPI (*blue*) in the UM and adjacent normal uveal tissues of clinical patients. (**B**) Western blotting analysis of PIEZO1 protein expression and quantification in 92.1 and Mel270 cells cultured on soft or stiff substrates, with GAPDH as a loading control (*n* = 3). (**C**) The qRT-PCR analysis of *PIEZO1* mRNA expression in 92.1 and Mel270 cells cultured on soft or stiff substrates (*n* = 3). (**D**) Western blotting analysis of PIEZO1 protein expression and quantification in tumor tissues from BAPN- and NS-treated groups (*n* = 5). Data are presented as mean ± SD. **P < 0.01, ***P < 0.001.

Immunofluorescence analysis of clinical UM specimens revealed that PIEZO1 expression was significantly higher in tumor tissues than in adjacent normal uveal tissues (Fig. 3A). This suggests a potential role for PIEZO1 in UM pathogenesis. To examine whether ECM stiffness regulates PIEZO1 expression, 92.1 and Mel270 cells were cultured on PAAGs with varying stiffnesses. Stiff substrates upregulated PIEZO1 expression, as evidenced by increases at both the mRNA level (2.3-fold in 92.1 cells and 2.5-fold in Mel270 cells) and protein level (1.6-fold in 92.1 cells and 1.5-fold in Mel270 cells) in vitro (Figs. 3B, 3C), which was further confirmed by immunofluorescence staining (Supplementary Fig. S6A). Conversely, BAPN-induced ECM softening reduced PIEZO1 protein levels by 55% in vivo (Fig. 3D). These results indicate that ECM stiffness regulates PIEZO1 expression both in vitro and in vivo.

**Figure 4.**
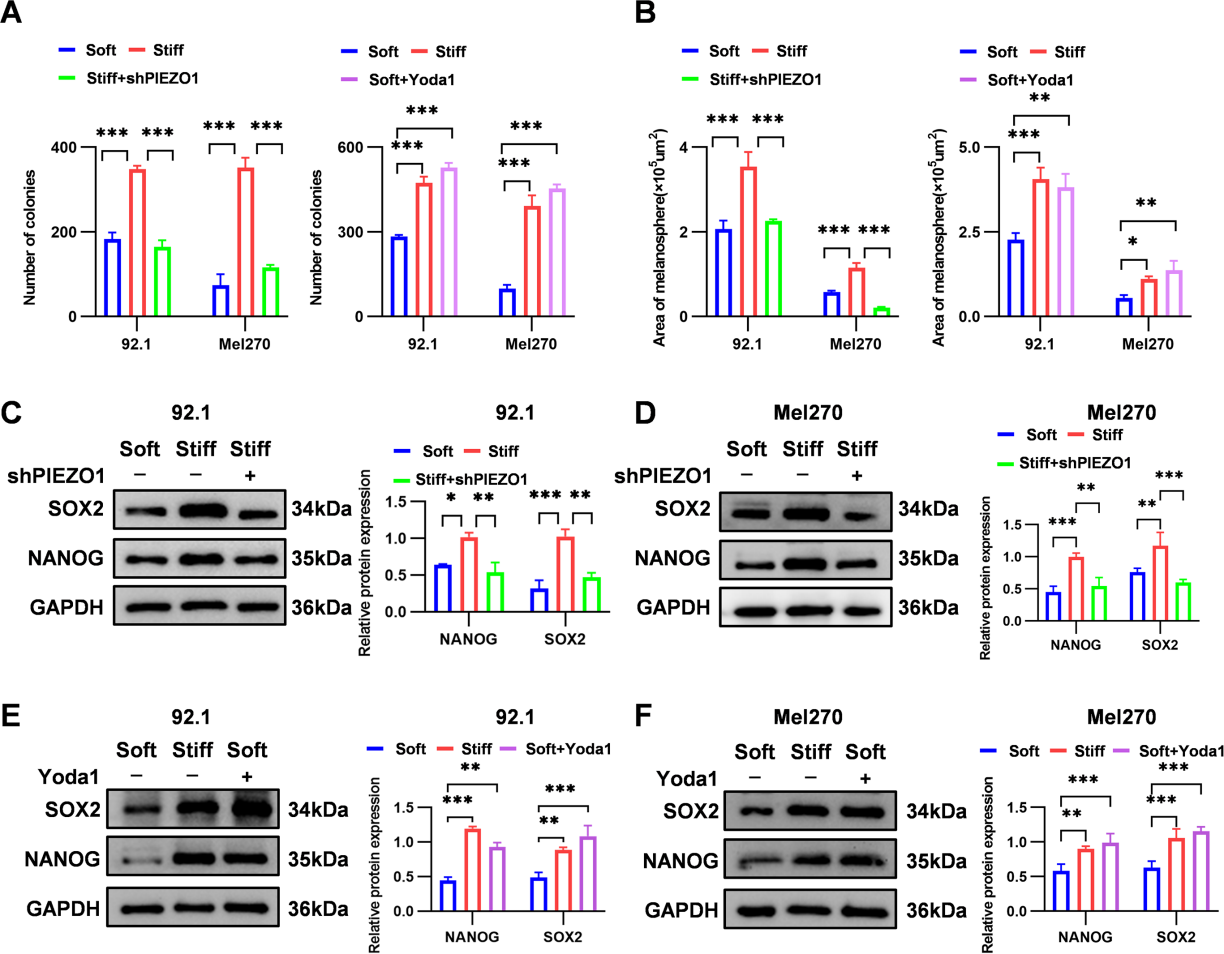
ECM stiffness regulates UM stemness via PIEZO1. (**A**) Quantification of colony formation in 92.1 and Mel270 cells following *PIEZO1* knockdown under stiff-substrate conditions (*left*) and PIEZO1 activation with Yoda1 under soft-substrate conditions (*right*). (**B**) Quantification of tumorsphere area in 92.1 and Mel270 cells after *PIEZO1* knockdown on stiff substrates (*left*) and PIEZO1 activation on soft substrates (*right*). (**C**, **D**) Western blotting analysis and quantification of NANOG and SOX2 protein expression in 92.1 (**C**) and Mel270 (**D**) cells after *PIEZO1* knockdown under stiff-substrate conditions. GAPDH was used as a loading control. (**E**, **F**) Western blotting analysis and quantification of NANOG and SOX2 protein expression in 92.1 cells (**E**) and Mel270 cells (**F**) treated with Yoda1 on soft substrates, with GAPDH as a loading control. Data are presented as mean ± SD (*n* = 3). *P < 0.05, **P < 0.01, ***P < 0.001.

We established stable *PIEZO1*-knockdown 92.1 and Mel270 cells (sh*PIEZO1*) and activated PIEZO1 pharmacologically with Yoda1 to determine whether ECM stiffness regulates UM stemness through PIEZO1. *PIEZO1* knockdown significantly reduced PIEZO1 mRNA and protein levels, whereas Yoda1 treatment increased PIEZO1 protein expression and calcium influx, confirming effective pathway activation (Supplementary Figs. S7A–S7F). Under stiff-substrate conditions, *PIEZO1* knockdown suppressed stemness phenotypes, reducing colony formation (53% in 92.1 cells and 67% in Mel270 cells) and tumorsphere area (36% in 92.1 cells and 82% in Mel270 cells) (Figs. 4A, 4B; Supplementary Figs. S8A, S8C). Conversely, its activation on soft substrates enhanced colony formation (1.9-fold in 92.1 cells and 4.6-fold in Mel270 cells) and tumorsphere area (1.7-fold in 92.1 cells and 2.5-fold in Mel270 cells) (Figs. 4A, 4B; Supplementary Figs. S8B, S8D). Molecular studies revealed that *PIEZO1* knockdown on stiff substrates significantly downregulated NANOG (47% in 92.1 cells and 46% in Mel270 cells) and SOX2 (54% in 92.1 cells and 41% in Mel270 cells) protein levels compared with shNC controls (Figs. 4C, 4D). These findings were further confirmed by immunofluorescence staining (Supplementary Figs. S6B, S6C). Conversely, Yoda1 treatment on soft substrates upregulated NANOG (2.0-fold in 92.1 cells and 1.7-fold in Mel270 cells) and SOX2 (2.2-fold in 92.1 cells and 1.8-fold in Mel270 cells) compared with soft-substrate controls (Figs. 4E, 4F). These findings suggest that PIEZO1 serves as an effector molecule in ECM stiffness-mediated regulation of UM stemness.

### ECM Stiffness Regulates UM Stemness via DOT1L

**Figure 5.**
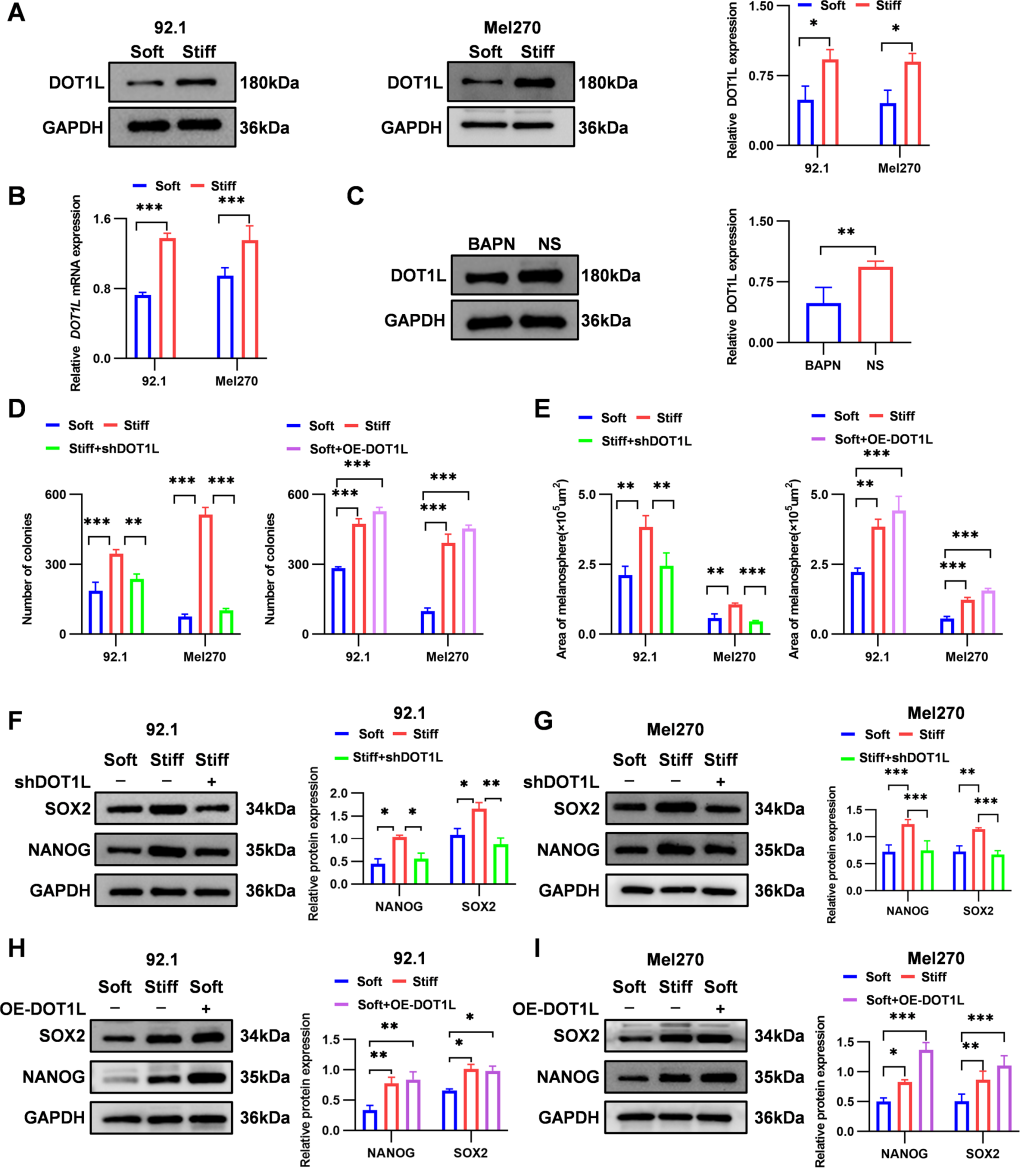
ECM stiffness regulates UM stemness through DOT1L. (**A**) Western blotting analysis and quantification of DOT1L protein expression in 92.1 and Mel270 cells cultured on soft and stiff substrates, with GAPDH as the loading control (*n* = 3). (**B**) The qRT-PCR analysis and quantification of *DOT1L* mRNA expression in 92.1 and Mel270 cells cultured on soft or stiff substrates (*n* = 3). (**C**) Western blotting analysis of DOT1L protein expression and quantification in tumor tissues from BAPN- and NS-treated groups (*n* = 5). (**D**) Quantification of colony formation in 92.1 and Mel270 cells following *DOT1L* knockdown under stiff-substrate conditions (*left*) and *DOT1L* overexpressing (OE-*DOT1L*) under soft-substrate conditions (*right*) (*n* = 3). (**E**) Quantification of tumorsphere area in 92.1 cells (*left*) and Mel270 cells (*right*) after *DOT1L* knockdown on stiff substrates (*left*) and OE-*DOT1L* on soft substrates (*right*) (*n* = 3). (**F**, **G**) Western blotting analysis and quantification of NANOG and SOX2 protein expression in 92.1 cells (**F**) and Mel270 cells (**G**) after *DOT1L* knockdown under stiff-substrate conditions(*n* = 3). (**H**, **I**) Western blotting analysis and quantification of NANOG and SOX2 protein expression in 92.1 cells (**H**) and Mel270 cells (**I**) after OE-*DOT1L* on soft substrates(*n* = 3). Data are presented as mean ± SD. *P < 0.05, **P < 0.01, ***P < 0.001.

We further explored the role of DOT1L in ECM stiffness-mediated regulation of UM stemness. DOT1L expression was upregulated by stiff substrates in vitro, with increases in mRNA (1.9-fold in 92.1 cells and 1.4-fold in Mel270 cells) and protein (1.9-fold in 92.1 cells and 2.0-fold in Mel270 cells) levels (Figs. 5A, 5B), as confirmed by immunofluorescence (Supplementary Fig. S9A). Corroborating this, ECM softening in vivo reduced DOT1L protein expression by 48% (Fig. 5C).

We also constructed 92.1 and Mel270 cells with stable knockdown (sh*DOT1L*) and overexpression (OE-*DOT1L*) of *DOT1L*. DOT1L mRNA and protein levels were effectively decreased in the sh*DOT1L* group and markedly increased in the OE-*DOT1L* group (Supplementary Figs. S7G–S7L). We found that *DOT1L* knockdown on stiff substrates markedly impaired stemness, reducing colony formation (31% in 92.1 cells and 80% in Mel270 cells) and tumorsphere area (36% in 92.1 cells and 57% in Mel270 cells) (Figs. 5D, 5E; Supplementary Figs. S10A, S10C). Conversely, *DOT1L* overexpression on soft substrates significantly enhanced both colony formation (1.9-fold in 92.1 cells and 4.6-fold in Mel270 cells) and tumorsphere expansion (2.0-fold in 92.1 cells and 2.8-fold in Mel270 cells) (Figs. 5D, 5E; Supplementary Fig. S10B, S10D). At the molecular level, these phenotypic changes were accompanied by corresponding alterations in stemness markers. *DOT1L* knockdown on stiff substrates downregulated both NANOG (46% in 92.1 cells and 40% in Mel270 cells) and SOX2 (47% in 92.1 cells and 41% in Mel270 cells) (Figs. 5F, 5G), These findings were further confirmed by immunofluorescence staining (Supplementary Figs. S9B, S9C). Conversely, *DOT1L* overexpression on soft substrates upregulated NANOG (2.5-fold in 92.1 cells and 2.7-fold in Mel270 cells) and SOX2 (1.5-fold in 92.1 cells and 2.2-fold in Mel270 cells) (Figs. 5H, 5I). These data indicate that DOT1L regulates NANOG and SOX2 expression in a stiffness-dependent manner in UM cells.

### ECM Stiffness Regulates DOT1L Expression via PIEZO1

**Figure 6.**
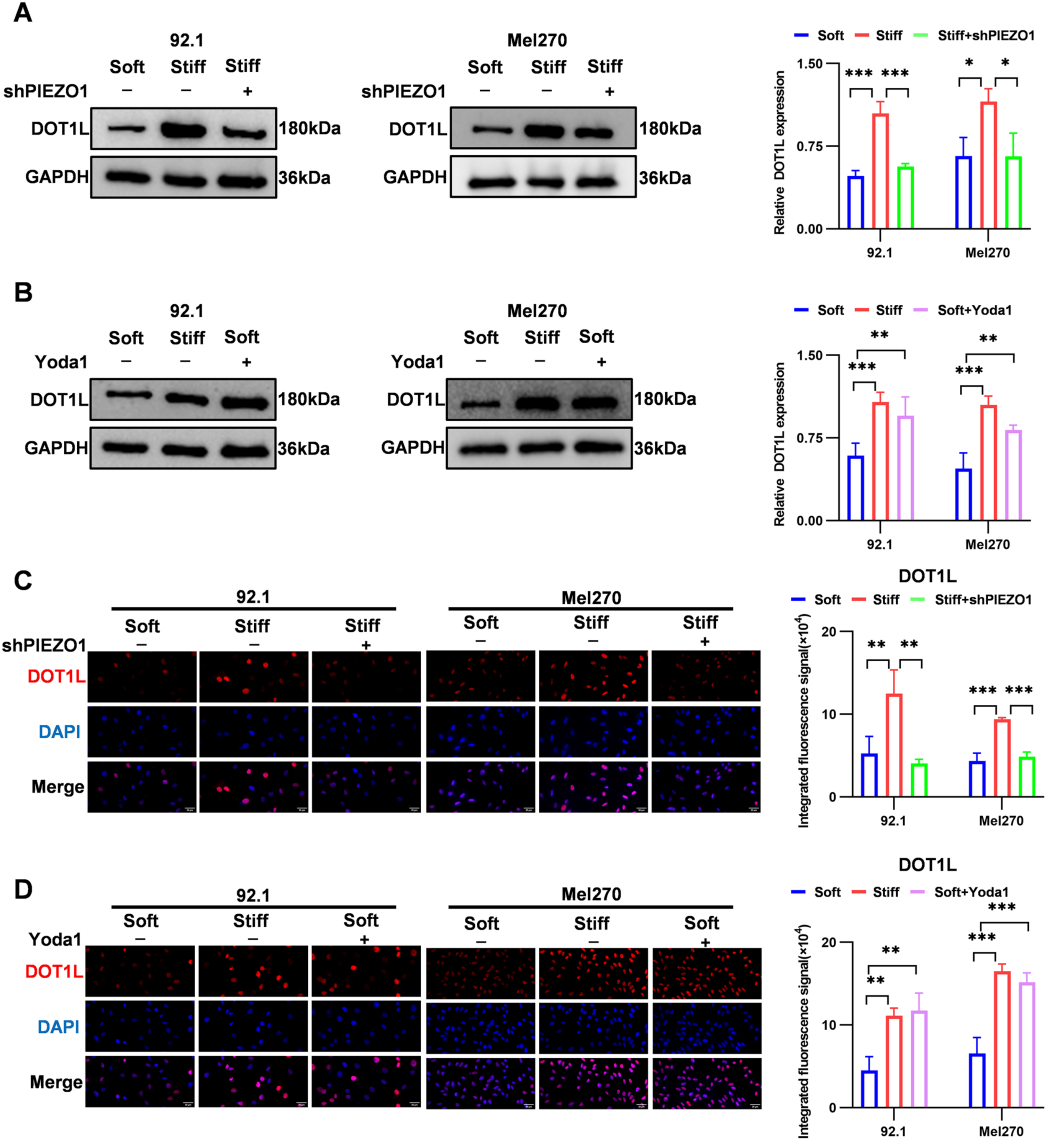
ECM stiffness regulates DOT1L expression through PIEZO1. (**A**) Western blotting analysis and quantification of DOT1L protein expression in 92.1 and Mel270 cells under stiff-substrate conditions following *PIEZO1* knockdown using GAPDH as a loading control. (**B**) Western blotting analysis and quantification of DOT1L protein expression in 92.1 and Mel270 cells cultured on soft substrates following Yoda1 treatment, using GAPDH as a loading control. (**C**) Immunofluorescence analysis of DOT1L protein (*red*) and DAPI (*blue*) in 92.1 and Mel270 cells under stiff-substrate conditions after *PIEZO1* knockdown. *Scale* *bar*: 30 µm. (**D**) Immunofluorescence analysis of DOT1L protein (*red*) and DAPI (*blue*) in 92.1 and Mel270 cells cultured on soft substrates after Yoda1 treatment. *Scale bar*: 30 µm. Data are presented as mean ± SD (*n* = 3). *P < 0.05, **P < 0.01, ***P < 0.001.

The above-mentioned experimental results reveal that ECM stiffness enhances UM stemness through PIEZO1 and upregulates the expression of the stemness markers (NANOG/SOX2) via DOT1L. A new question arises as to whether these two mechanisms form a regulatory network that jointly determines UM stemness. To this end, we explored whether PIEZO1 regulates DOT1L expression in response to ECM stiffness. Western blotting showed that *PIEZO1* knockdown under stiff-substrate conditions reduced DOT1L expression by 46% in 92.1 cells and 57% in Mel270 cells (Fig. 6A). Conversely, PIEZO1 activation with Yoda1 under soft-substrate conditions increased DOT1L levels by 1.6-fold in 92.1 cells and 1.7-fold in Mel270 cells (Fig. 6B). Immunofluorescence analysis confirmed this regulatory relationship (Figs. 6C, 6D). These findings demonstrate that ECM stiffness positively regulates DOT1L expression via PIEZO1, suggesting that PIEZO1 and DOT1L form a mechanotransduction cascade to cooperatively drive UM stemness.

### PIEZO1–DOT1L Axis Regulates UM Stemness In Vivo

**Figure 7.**
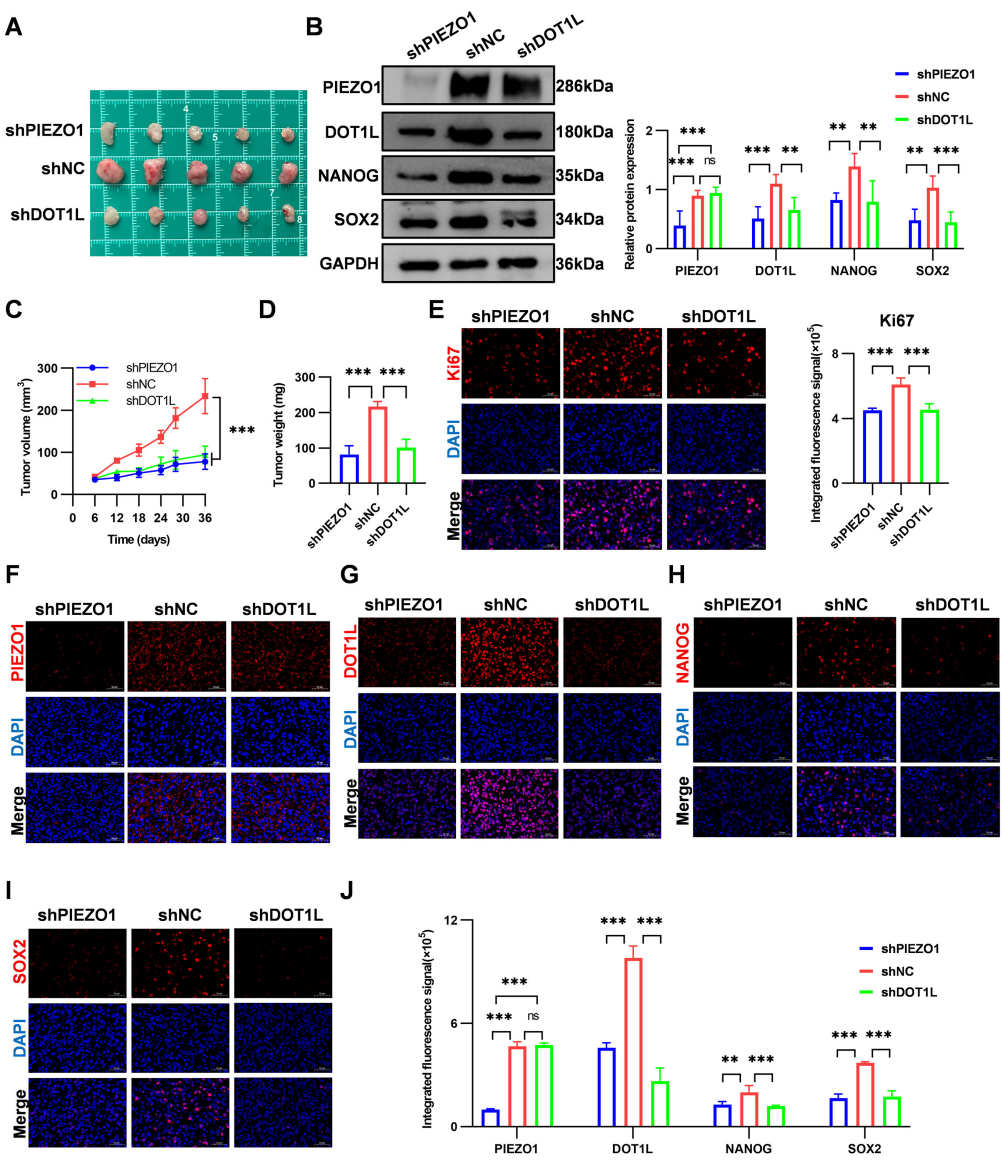
Mechanism of PIEZO1 and DOT1L in regulating UM stemness in vivo. (**A**) Images of subcutaneous tumors in the sh*PIEZO1*, shNC, and sh*DOT1L* groups. (**B**) Western blotting analysis and quantification of PIEZO1, DOT1L, NANOG, and SOX2 protein expression in sh*PIEZO1*, shNC, and sh*DOT1L* groups, with GAPDH as a loading control. (**C**) Tumor growth curves in the shNC, sh*PIEZO1*, and sh*DOT1L* groups. (**D**) Quantification of tumor weights in the sh*PIEZO1*, shNC, and sh*DOT1L* groups. (**E**) Immunofluorescence analysis of Ki67 (*red*) with nuclear counterstain DAPI (*blue*) in tumor tissues from sh*PIEZO1*, shNC, and sh*DOT1L* treatment groups. *Scale bar*: 50 µm. (**F**–**I**) Immunofluorescence images of PIEZO1 (**F**), DOT1L (**G**), NANOG (**H**), and SOX2 (**I**) protein expression (*red*) with nuclear counterstain DAPI (*blue*) in tumor tissues from sh*PIEZO1*, shNC, and sh*DOT1L* treatment groups. *Scale bar*: 50 µm. (**J**) Quantification of PIEZO1-, DOT1L-, NANOG-, and SOX2-positive signals corresponding to panels **F** to **I**. Data are presented as mean ± SD (*n* = 5). **P < 0.01, ***P < 0.001; ns, not significant (*P* > 0.05).

We established a subcutaneous xenograft model using stable knockdown UM cell lines to evaluate the in vivo role of the PIEZO1–DOT1L axis in UM stemness. The experimental groups included control (shNC), *PIEZO1* knockdown (sh*PIEZO1*), and *DOT1L* knockdown (sh*DOT1L*). No significant differences were observed in the body weight of the mice among the groups (Supplementary Fig. S3B), indicating that *PIEZO1* or *DOT1L* knockdown did not affect overall health or systemic growth. Both knockdown groups exhibited markedly inhibited tumor growth compared with the control group. Tumor volume decreased by 66% in the sh*PIEZO1* group and 60% in the sh*DOT1L* group, with corresponding reductions in tumor weight of 62% and 53% (Figs. 7A, 7C, 7D). This was accompanied by reduced proliferation, with Ki-67–positive cells decreasing by 32% and 27% in the sh*PIEZO1* and sh*DOT1L* groups, respectively (Fig. 7E). At the molecular level, western blotting and immunofluorescence further revealed that NANOG and SOX2 protein expression was reduced in both knockdown groups (*P* < 0.05) (Figs. 7B, 7F–J), indicating impaired stemness. Immunofluorescence confirmed stable expression of melanocytic marker Melan-A across all groups (Supplementary Fig. S5B), indicating that *PIEZO1* or *DOT1L* knockdown did not alter the lineage identity of the tumor cells. Mechanistically, *PIEZO1* knockdown decreased both PIEZO1 and DOT1L protein levels, while *DOT1L* knockdown selectively reduced DOT1L without altering PIEZO1 expression (Figs. 7B, 7F–J). This suggests that PIEZO1 acts upstream of DOT1L. Overall, these findings provide important in vivo evidence that ECM stiffness promotes UM stemness through the PIEZO1–DOT1L signaling axis.

### Clinical Relevance of PIEZO1 and DOT1L Expression in UM

Analysis of the TCGA–UM cohort confirmed the clinical relevance of *PIEZO1* and *DOT1L*. Kaplan–Meier analysis showed that high expression of either *PIEZO1* (hazard ratio [HR] = 1.53, *P* < 0.001) or *DOT1L* (HR = 2.17, *P* = 0.001) was associated with poorer overall survival (Supplementary Fig. S11A, S11B), further highlighting the clinical relevance of the PIEZO1–DOT1L axis in UM.

## Discussion

**Figure 8.**
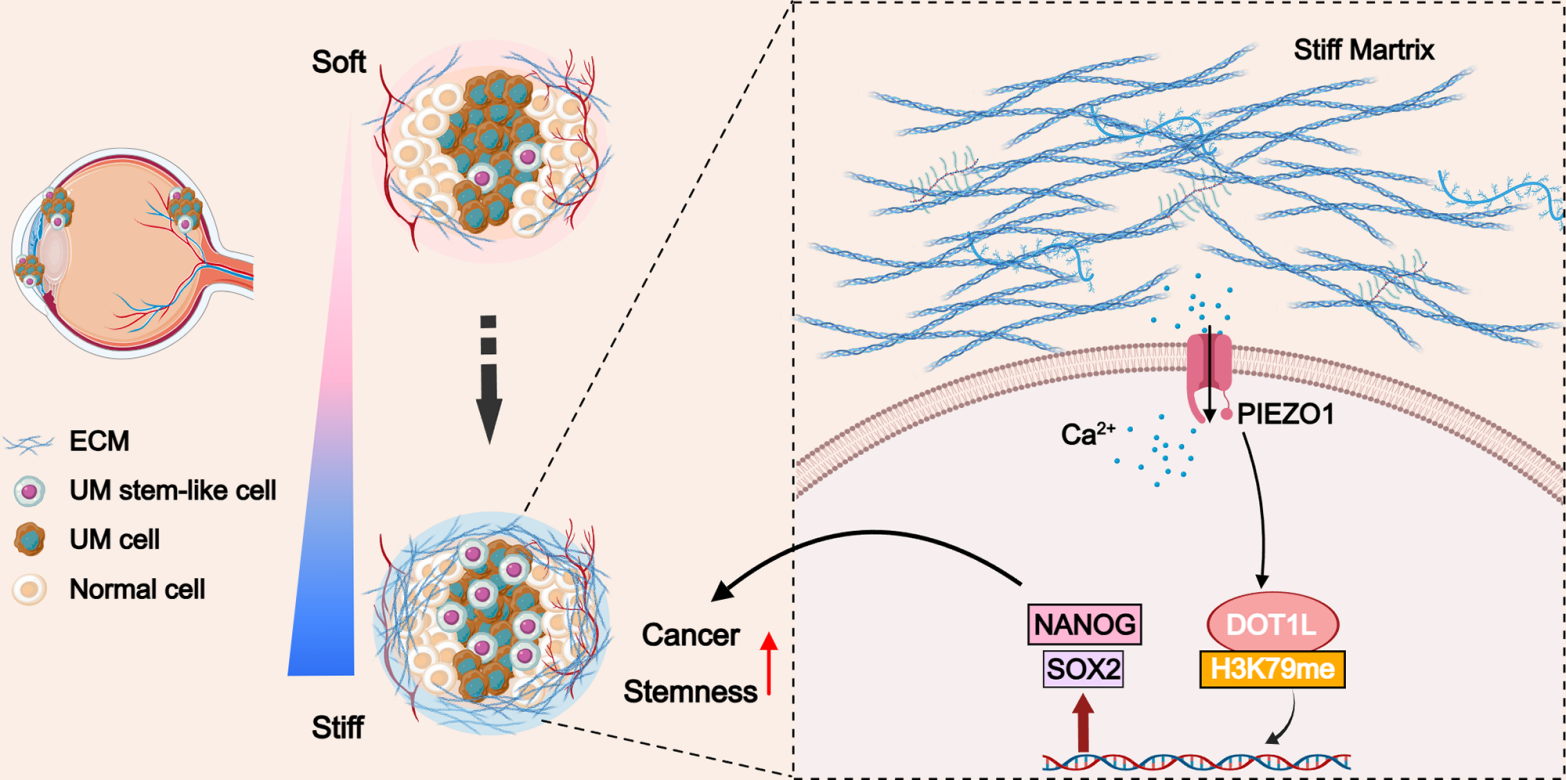
Diagram illustrating the role of the PIEZO1–DOT1L axis in ECM stiffness–induced stemness and tumor progression in UM.

Current therapeutic strategies targeting CSCs primarily focus on modulating the TME that maintains cancer stemness,^9^^,^^14^^,^^41^ blocking stemness-related signaling pathways,^42^^,^^43^ or directly targeting CSC surface markers.^44^ However, TME-mediated regulation of CSCs in UM has been rarely investigated. To our knowledge, this study provides the first evidence that elevated ECM stiffness enhanced UM stemness. Mechanistic studies demonstrated that PIEZO1 regulates DOT1L by sensing ECM stiffness changes, subsequently driving NANOG and SOX2 expression and promoting UM stemness (Fig. 8). We further revealed that increased ECM stiffness induces chemoresistance, aligning with observations in breast cancer, where ECM stiffness promotes drug resistance.^11^ These results indicate that targeting the mechanical microenvironment may provide novel therapeutic strategies for UM. Importantly, high expression of *PIEZO1* and *DOT1L* correlates with poor patient survival, highlighting the clinical relevance of this mechanotransduction axis.

Although previous studies on PIEZO1 have largely focused on tumor apoptosis and metastasis, its role in regulating cancer stemness has been less examined.^22^^,^^25^^,^^45^^,^^46^ The core innovation of this study was to reveal, for the first time, to our knowledge, the critical role of PIEZO1 in regulating UM stemness. We found that PIEZO1 expression increases in response to elevated ECM stiffness using both in vitro and in vivo models. Functionally, *PIEZO1* knockdown abolished stiffness-induced enhancement of stemness, whereas pharmacological activation with Yoda1 restored stemness under soft-matrix conditions. Consistent with previous reports in prostate cancer, osteosarcoma, and glioblastoma,^23^^,^^26^^,^^47^^,^^48^ our findings collectively demonstrate that mechanical signaling promotes malignant tumor progression via PIEZO1. These results suggest that targeting PIEZO1 could be a novel therapeutic approach for UM.

DOT1L, as a pivotal epigenetic factor regulating cancer stemness, is an emerging therapeutic target for various cancers.^13^^,^^29^^,^^30^^,^^49^ Previous studies have focused on DOT1L regulation via biochemical signaling. For example, Bourguignon et al.^30^ revealed that the aberrant HA deposition activates DOT1L through CD44 to enhance stemness in head and neck cancer. Meanwhile, Kryczek et al.^13^ demonstrated that interleukin‑22 upregulates NANOG and SOX2 expression through the STAT3–DOT1L axis to promote colorectal cancer stemness. Our study revealed how mechanical signals regulate DOT1L. We found that ECM stiffness upregulates DOT1L to drive NANOG and SOX2 expression, thereby enhancing UM stemness. Together with published findings, our data suggest that DOT1L may serve as a central integrator of mechano-biochemical signaling within the TME. We further demonstrated that PIEZO1 promotes DOT1L expression by sensing ECM stiffness changes. Prior work in glioma showed that stiffness-induced PIEZO1 activation triggers integrin–focal adhesion kinase (FAK) signaling, thereby enhancing ECM synthesis and HA deposition in a positive feedback loop that amplifies tissue stiffness.^23^ Accordingly, we hypothesized that a mechano-biochemical synergistic regulatory network exists in UM as follows: (1) Via a mechanical signaling axis, ECM stiffness directly activates DOT1L through PIEZO1-mediated mechanotransduction; and (2) via a biochemical signaling axis, PIEZO1‑induced HA deposition activates DOT1L through the CD44‑dependent pathway. This mechano-biochemical integrated regulatory model provides a new perspective on targeting interventions within the TME. Nonetheless, the precise molecular pathways involved require further experimental validation.

Our findings identify the PIEZO1–DOT1L axis as a stiffness-responsive pathway driving stemness in UM. This finding adds to a broader repertoire of stiffness-responsive mechanisms described in other cancers. For example, integrin-mediated YAP/TAZ activation transduces stiffness cues to regulate stemness transcriptional programs and CSC plasticity in gastric cancer and hepatocellular carcinoma.^12^^,^^50^ Stiffness-dependent integrin–discoidin domain receptor (DDR)–signal transducer and activator of transcription 1 (STAT1) signaling has also been shown to regulate dormancy–activation transitions in response to mechanical load.^16^ Stiffness additionally induces epigenetic reprogramming by upregulating KAT6A and enhancing H3K23 acetylation to activate SOX2.^18^ Furthermore, recent studies demonstrate that TAZ–NANOG condensates strengthen pluripotency networks under high ECM stiffness.^11^ Collectively, these studies illustrate that stiffness-driven stemness can arise from an integrated network encompassing diverse mechanosensitive, epigenetic, and biomolecular condensate-based mechanisms.

Our in vivo experiments demonstrated that BAPN-induced ECM softening significantly suppressed tumor growth by inhibiting the PIEZO1–DOT1L–NANOG/SOX2 axis. Recently, Shen et al.^41^ achieved synergistic regulation of the liver cancer microenvironment and intracellular signaling pathways through combined therapy with BAPN and siABHD11-AS1, markedly inhibiting tumor growth and metastasis. Zhu et al.^51^ reported that BAPN significantly enhanced drug permeability and antitumor efficacy in breast cancer treatment. Therefore, future studies can explore combination therapies involving BAPN with PIEZO1 (e.g., GsMTx4) and DOT1L (e.g., EPZ-5676) to overcome stiffness-associated drug resistance in UM. However, the synergistic efficacy and safety of this strategy require further experimental validation.

This study also has limitations. Although our data established a stiffness-responsive PIEZO1–DOT1L axis in UM, whether this mechanism similarly operates in other cancer contexts remains unknown. The therapeutic relevance of targeting PIEZO1 or DOT1L also requires further preclinical validation. Furthermore, we used only two UM cell lines and a single xenograft model, which may not fully represent the molecular heterogeneity of UM, particularly in terms of *GNAQ*/*GNA11* and BAP1 status.^52^ Additionally, each established model reflects only part of the diversity seen in patient tumors.^53^ Future work incorporating additional cell lines, and complementary in vivo models will be essential to evaluate the generalizability of this mechanotransduction pathway.

In conclusion, this study confirms that increased ECM stiffness upregulates DOT1L expression via PIEZO1, thereby promoting UM stemness. Our findings demonstrate that targeting ECM stiffness can effectively suppress UM stemness and suggest that PIEZO1 and DOT1L represent promising therapeutic targets for mitigating UM malignant progression.

## Supplementary Material

Supplement 1
