# Supplementary material for: Extracellular Matrix Stiffness Regulates Cancer Stemness in Uveal Melanoma via the PIEZO1–DOT1L Axis
Source: Invest Ophthalmol Vis Sci. 2025 Dec 18;66(15):57. doi: 10.1167/iovs.66.15.57 (PMC12716446; doi:10.1167/iovs.66.15.57)
Supplement: Supplement 1 [file iovs-66-15-57_s001.docx]

**Supplementary figure**

**
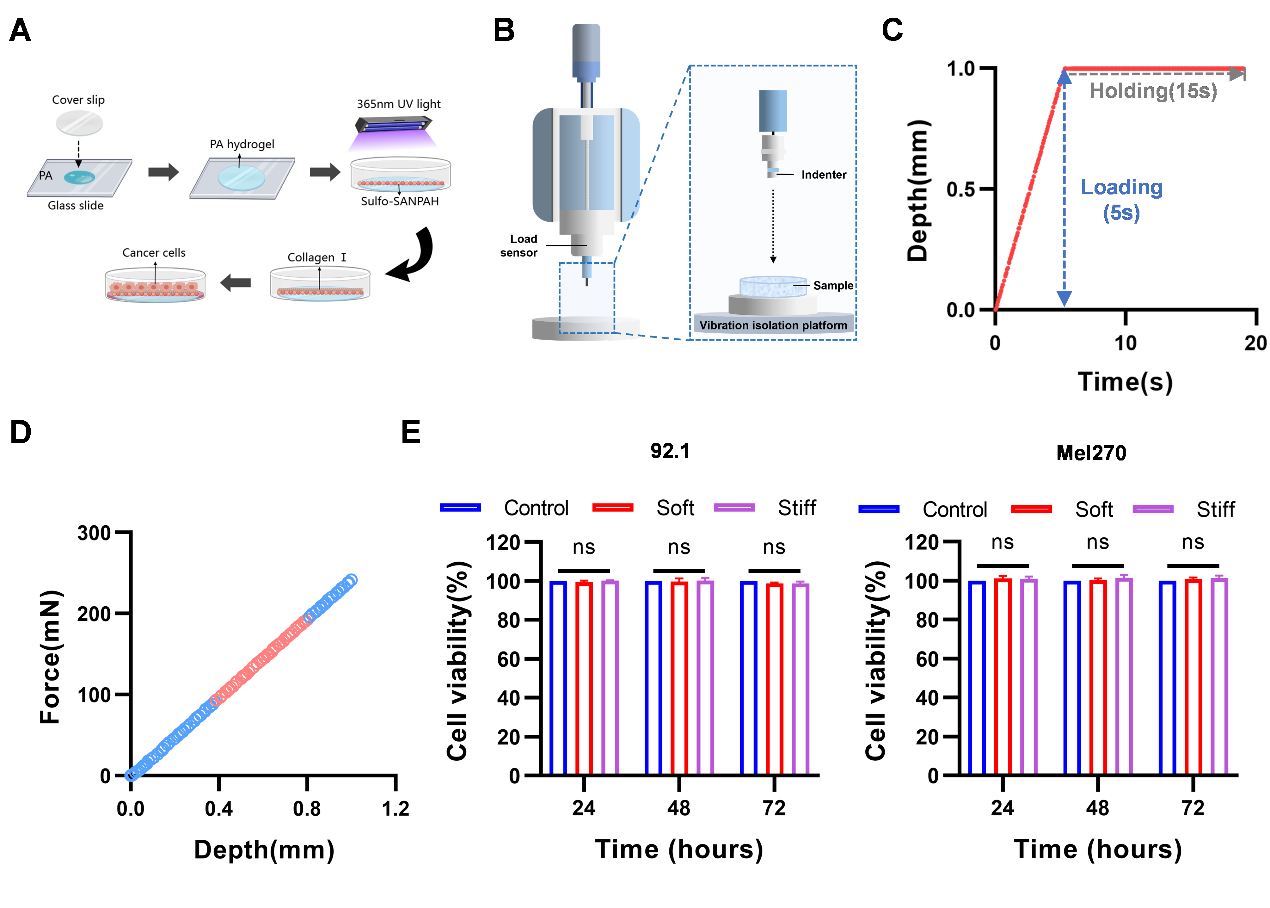
**

**Supplementary Figure S1.** Preparation and biocompatibility validation of PAAGs.

**(A)** Schematic of PAAGs preparation. **(B)** Measurement simulation diagram of the indentation test setup. **(C)** Depth-time curve during the indentation experiment. The test mode was indentation-relaxation mode, and the relevant test parameters were indentation time, relaxation time, and maximum indentation depth of 5s, 15s, and 1000 μm, respectively. **(D)** Available data used for the calculation of the elastic modulus. To eliminate the effect of nonlinearity in the initial contact phase, data in the interval of 40-80% of the indentation depth were selected for the elastic modulus calculation for the fitting analysis. Red data points were used for fitting and blue-colored data points were discarded. **(E)** Cytotoxicity of PAAGs was assessed using the CCK-8 method. Data are presented as mean ± SD (n = 3; ns: not significant, *P* > 0.05).


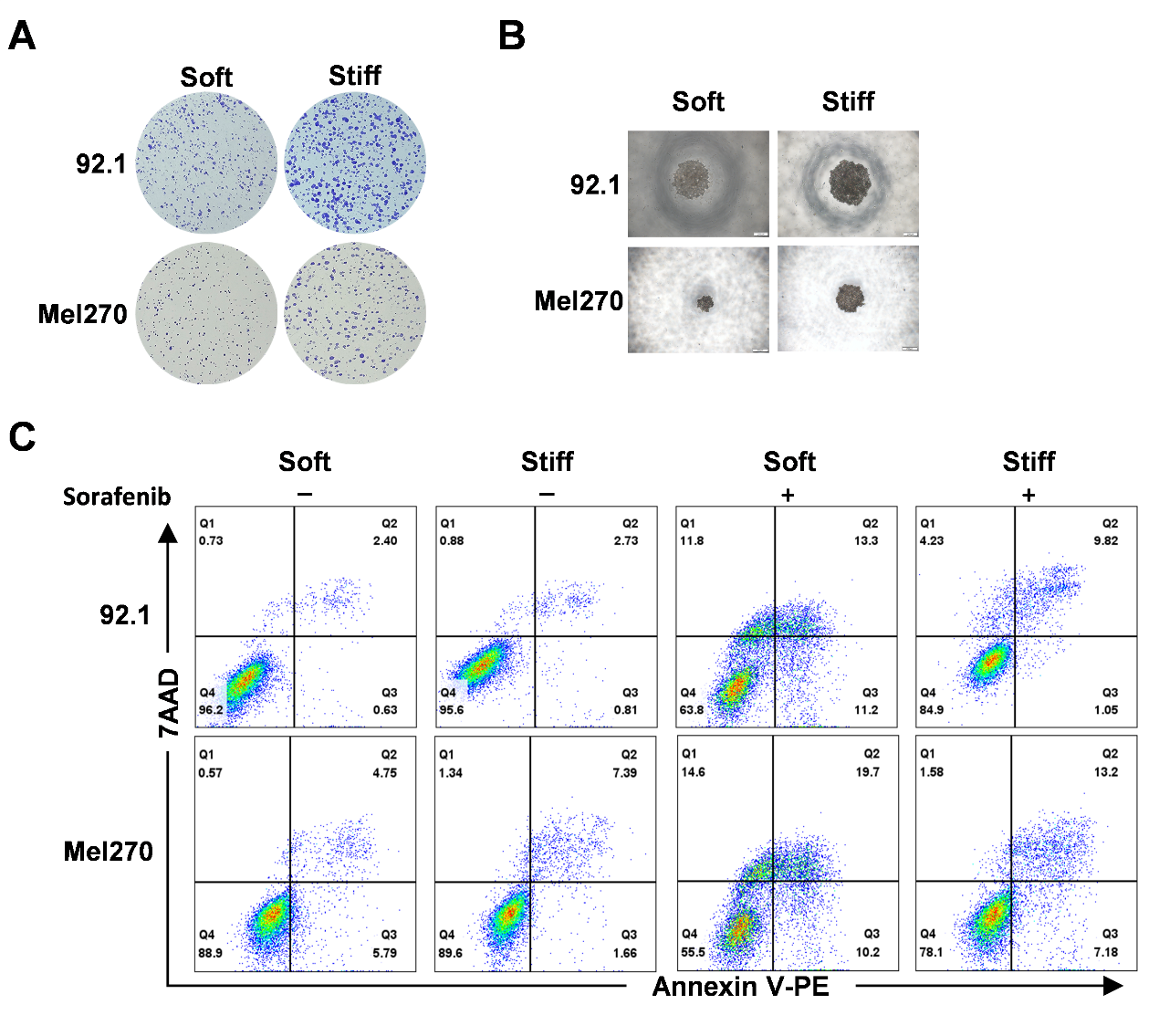


**Supplementary Figure S2.** Representative images of colony formation, tumorsphere formation, and apoptosis assays corresponding to quantifications in Figure 1. **(A)** Representative colony formation images of 92.1 and Mel270 cells cultured on soft and stiff substrates. **(B)** Representative tumorsphere formation images under soft and stiff conditions. **(C)** Flow cytometry plots showing Annexin V/PI–based apoptosis analysis in soft- and stiff-matrix cultures.


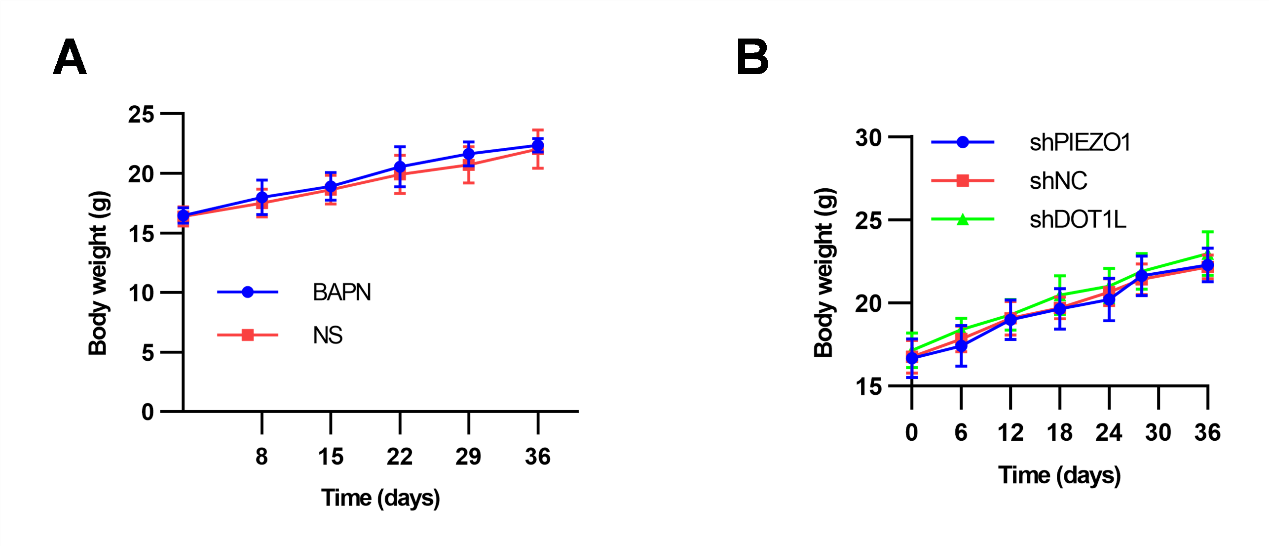


**Supplementary Figure S3.** Time-course monitoring of body weight in tumor-bearing mice. **(A)** Time course of body weight changes in nude mice treated with BAPN (soft ECM) or NS (stiff ECM) throughout the treatment period. **(B)** Body weight monitoring of mice bearing xenograft tumors derived from shNC, shPIEZO1, or shDOT1L cells during the experimental timeline.


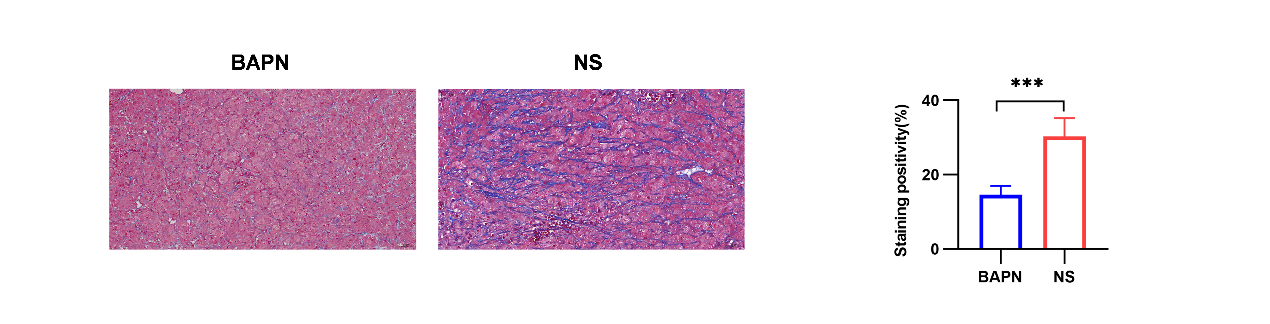


**Supplementary Figure S4.** Masson’s trichrome staining of tumor tissues from the BAPN-treated and NS control groups. Scale bar: 100 μm. Data are presented as mean ± SD (n = 5; ****P* < 0.001).


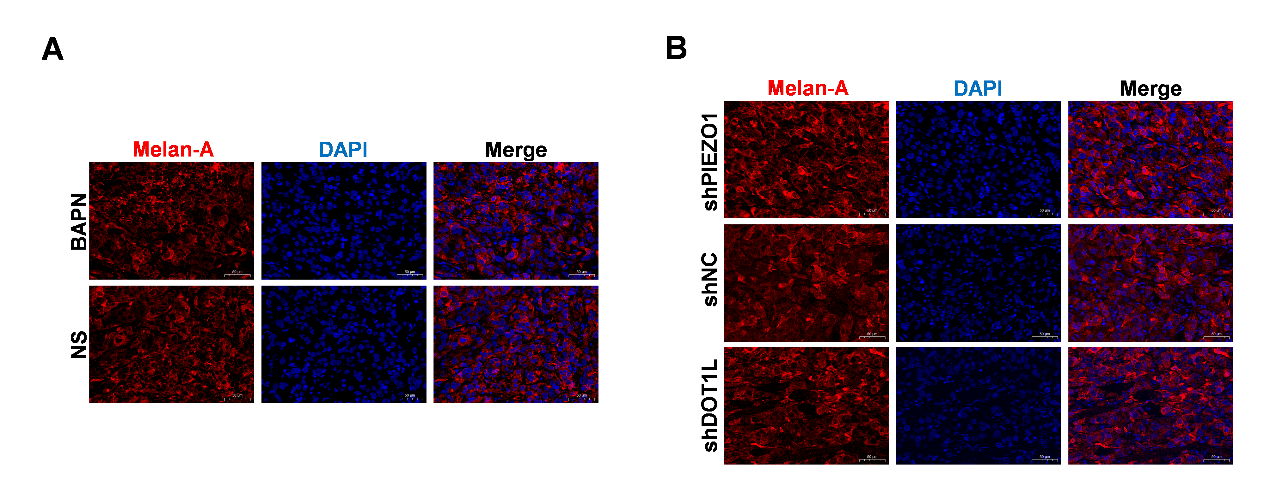


**Supplementary Figure S5.** Immunofluorescence staining of melanocytic marker in Mel270-derived uveal melanoma subcutaneous xenografts. **(A)** Immunofluorescence images of Melan-A protein (red) with DAPI nuclear counterstain (blue) in tumor tissues from the BAPN-treated and NS control groups. Scale bar: 50 μm. **(B)** Immunofluorescence images of Melan-A protein (red) with DAPI nuclear counterstain (blue) in tumor tissues from the shPIEZO1, shNC, and shDOT1L groups. Scale bar: 50 μm.


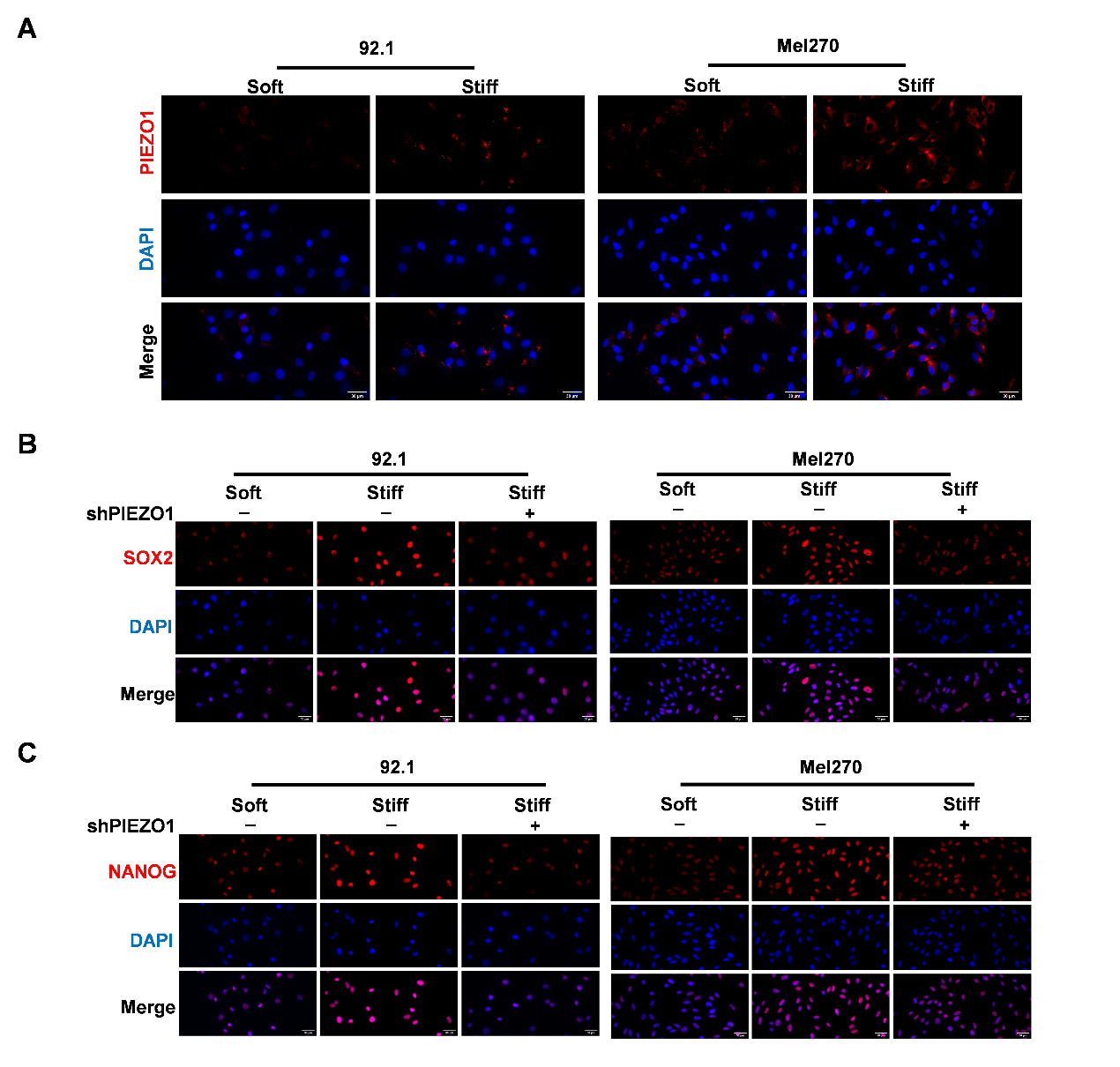


**Supplementary Figure S6.** Immunofluorescence analysis of PIEZO1 and stemness markers in UM cells under stiffness-regulated conditions. **(A)** Immunofluorescence images of PIEZO1 in 92.1 and Mel270 cells cultured on soft and stiff substrates. **(B)** Immunofluorescence staining of SOX2 in 92.1 and Mel270 cells following PIEZO1 knockdown under stiff-substrate conditions. **(C)** Immunofluorescence staining of NANOG in 92.1 and Mel270 cells after PIEZO1 knockdown under stiff-substrate conditions. Scale bar: 30 μm.


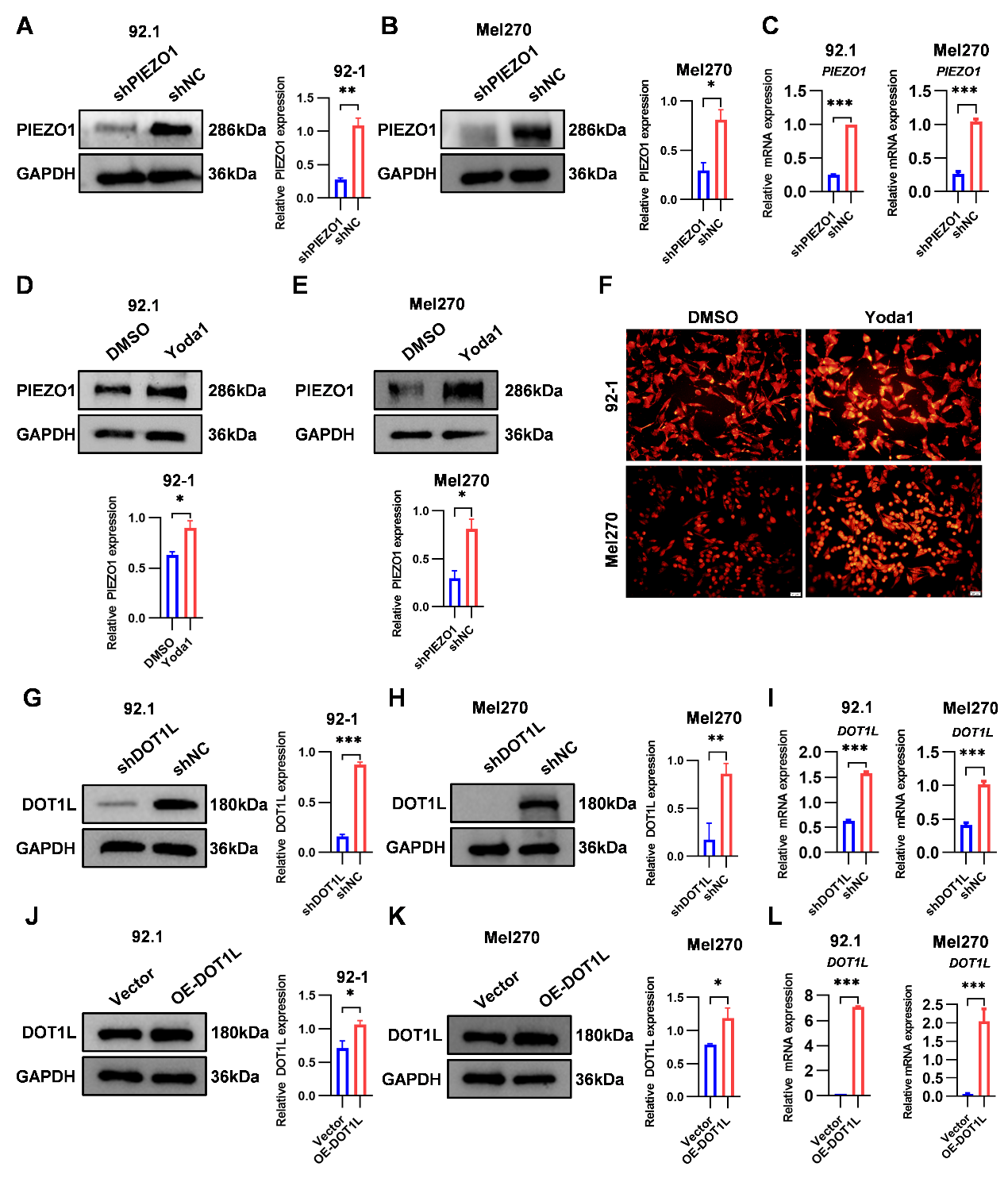


**Supplementary Figure S7.** Establishment and validation of stable cell lines. **(A-B)** Western blotting analysis and quantification of PIEZO1 protein knockdown in 92.1 (A) and Mel270 (B) cells transduced with shNC or shPIEZO1 lentivirus, using GAPDH as a loading control. **(C)** qRT-PCR analysis of PIEZO1 mRNA expression levels in 92.1 and Mel270 cells transduced with shNC or shPIEZO1 lentivirus. **(D-E)** Western blotting analysis and quantification of PIEZO1 protein expression in 92.1 (D) and Mel270 (E) cells treated with DMSO or Yoda1, using GAPDH as a loading control. **(F)** Calcium influx assay using the Rhod-2 AM probe in 92.1 and Mel270 cells treated with DMSO or Yoda1. **(G-H)** Western blotting analysis and quantification of DOT1L protein expression in 92.1 (G) and Mel270 (H) cells transduced with an shNC or shDOT1L lentivirus, using GAPDH as a loading control. **(I)** qRT-PCR analysis of DOT1L mRNA expression levels in 92.1 and Mel270 cells transduced with shNC or shDOT1L lentivirus. **(J-K)** Western blotting analysis and quantification of DOT1L overexpression in 92.1 (J) and Mel270 (K) cells transduced with empty vector (EV)- or DOT1L overexpression lentiviruses, using GAPDH as a loading control. **(L)** qRT-PCR analysis of DOT1L mRNA levels in 92.1 and Mel270 cells transduced with EV- or DOT1L overexpression lentiviruses. Data are presented as mean ± SD (n=5; **P* < 0.05; ***P* < 0.01; ****P* < 0.001).


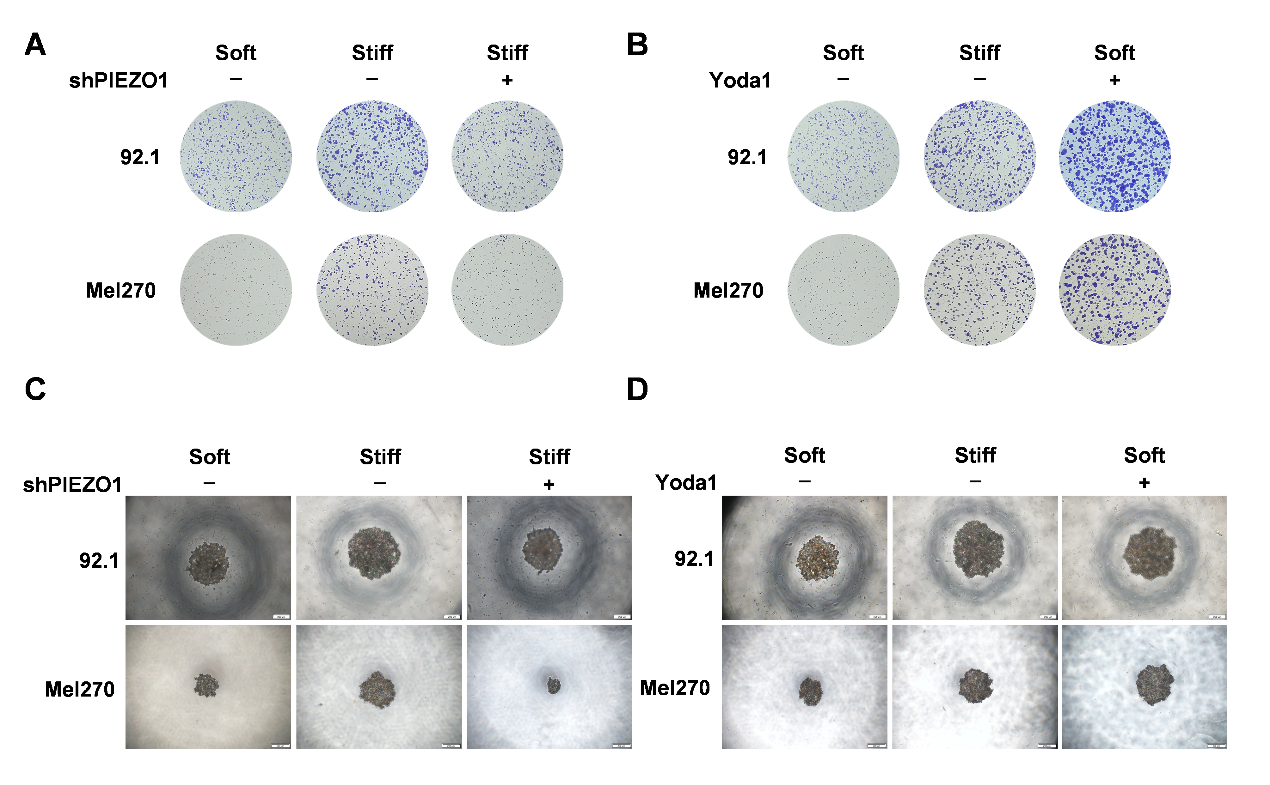


**Supplementary Figure S8.** Representative images of sphere formation and colony formation assays after PIEZO1 modulation in UM cells. **(A)** Representative tumorsphere images of 92.1 and Mel270 cells after PIEZO1 knockdown under stiff-substrate conditions. **(B)** Representative tumorsphere images of 92.1 and Mel270 cells following PIEZO1 activation with Yoda1 under soft-substrate conditions. **(C)** Representative colony formation images of 92.1 and Mel270 cells after PIEZO1 knockdown on stiff substrates. **(D)** Representative colony formation images of 92.1 and Mel270 cells following PIEZO1 activation on soft substrates. These images correspond to the quantitative analyses shown in Figure 4. Scale bar: 200 μm.


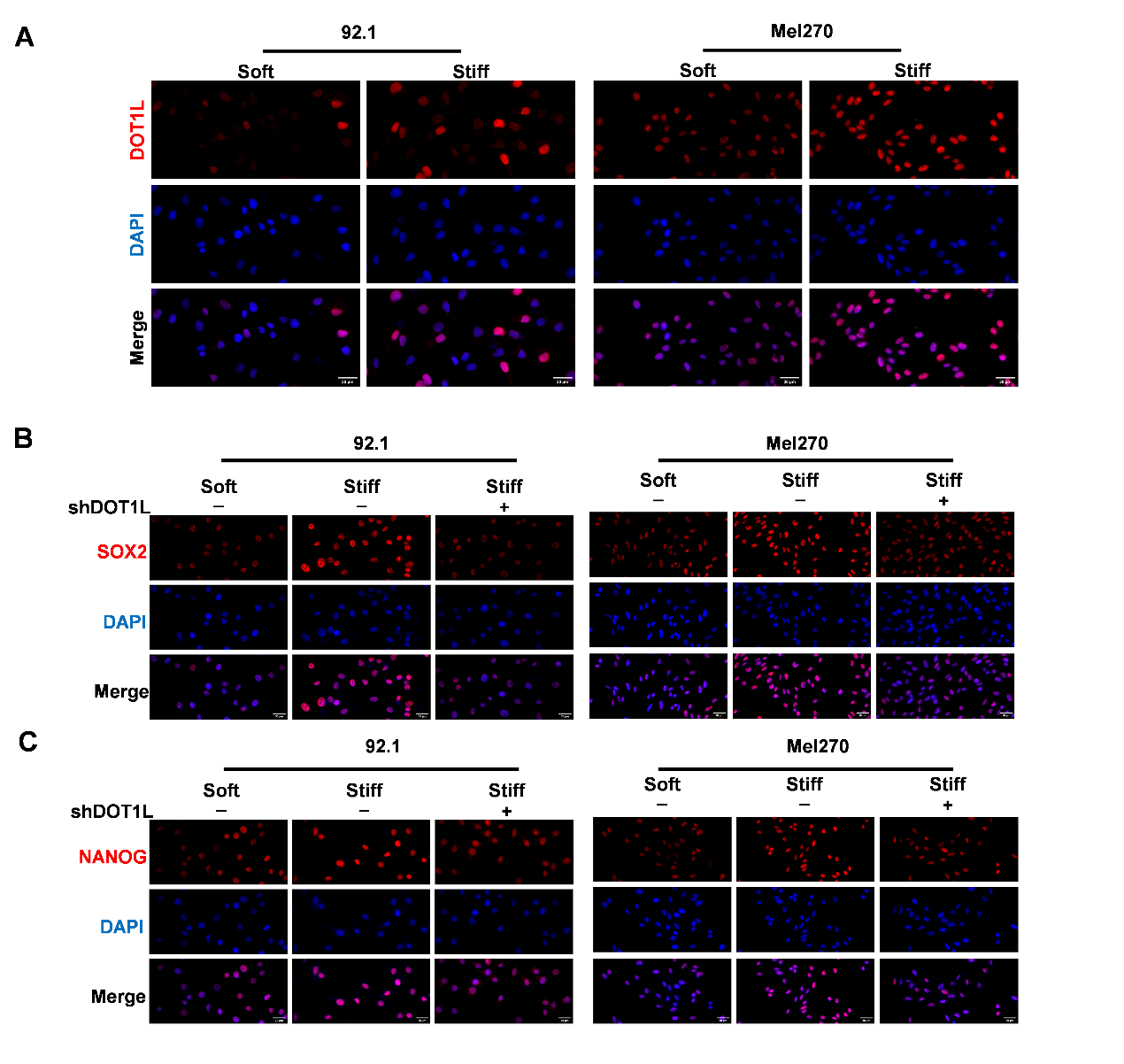


**Supplementary Figure S9.** Immunofluorescence analysis of DOT1L and stemness markers in UM cells under stiffness-regulated conditions. **(A)** Immunofluorescence images of DOT1L in 92.1 and Mel270 cells cultured on soft and stiff substrates. **(B)** Immunofluorescence staining of SOX2 in 92.1 and Mel270 cells following DOT1L knockdown under stiff-substrate conditions. **(C)** Immunofluorescence staining of NANOG in 92.1 and Mel270 cells after DOT1L knockdown under stiff-substrate conditions. Scale bar: 30 μm.


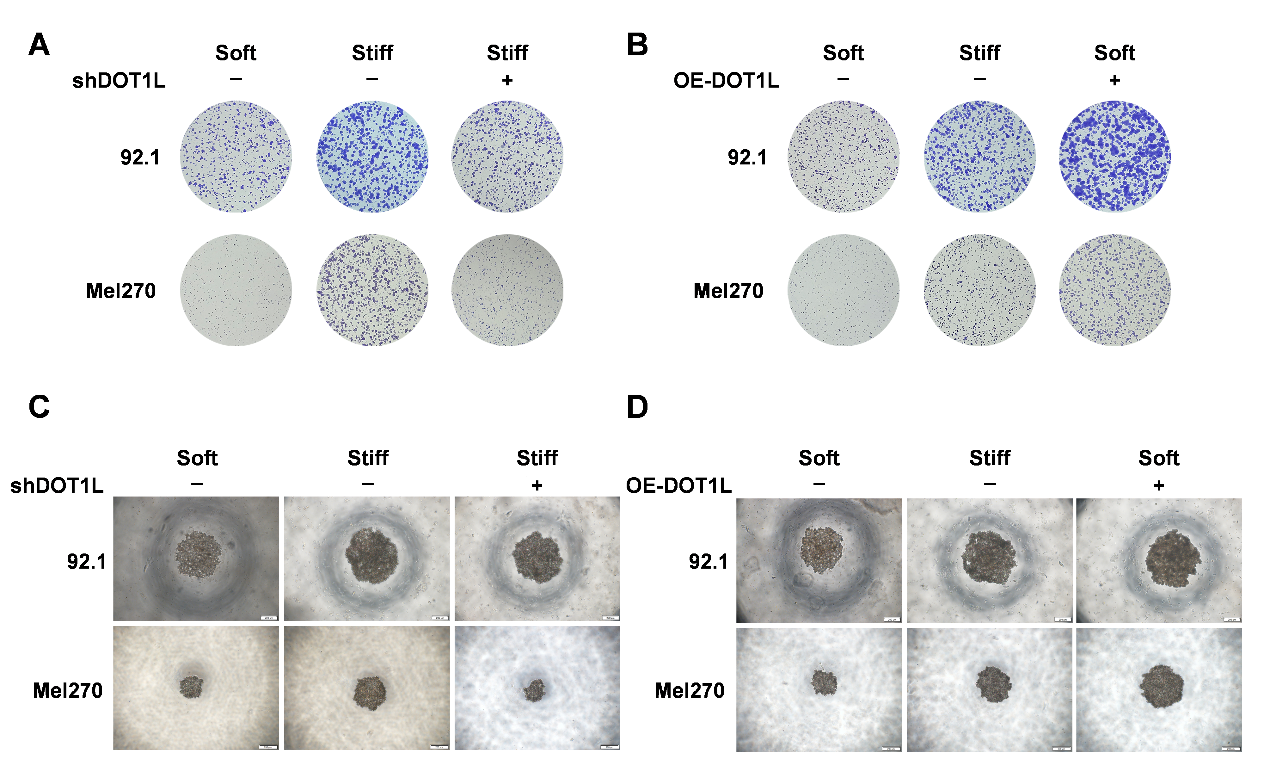


**Supplementary Figure S10.** Representative images of sphere formation and colony formation assays after DOT1L modulation in UM cells. **(A)** Representative tumorsphere images of 92.1 and Mel270 cells after DOT1L knockdown under stiff-substrate conditions. **(B)** Representative tumorsphere images of 92.1 and Mel270 cells following DOT1L overexpression under soft-substrate conditions. **(C)** Representative colony formation images of 92.1 and Mel270 cells after DOT1L knockdown on stiff substrates. **(D)** Representative colony formation images of 92.1 and Mel270 cells following DOT1L overexpression on soft substrates. These images correspond to the quantitative analyses shown in Figure 5. Scale bar: 200 μm.


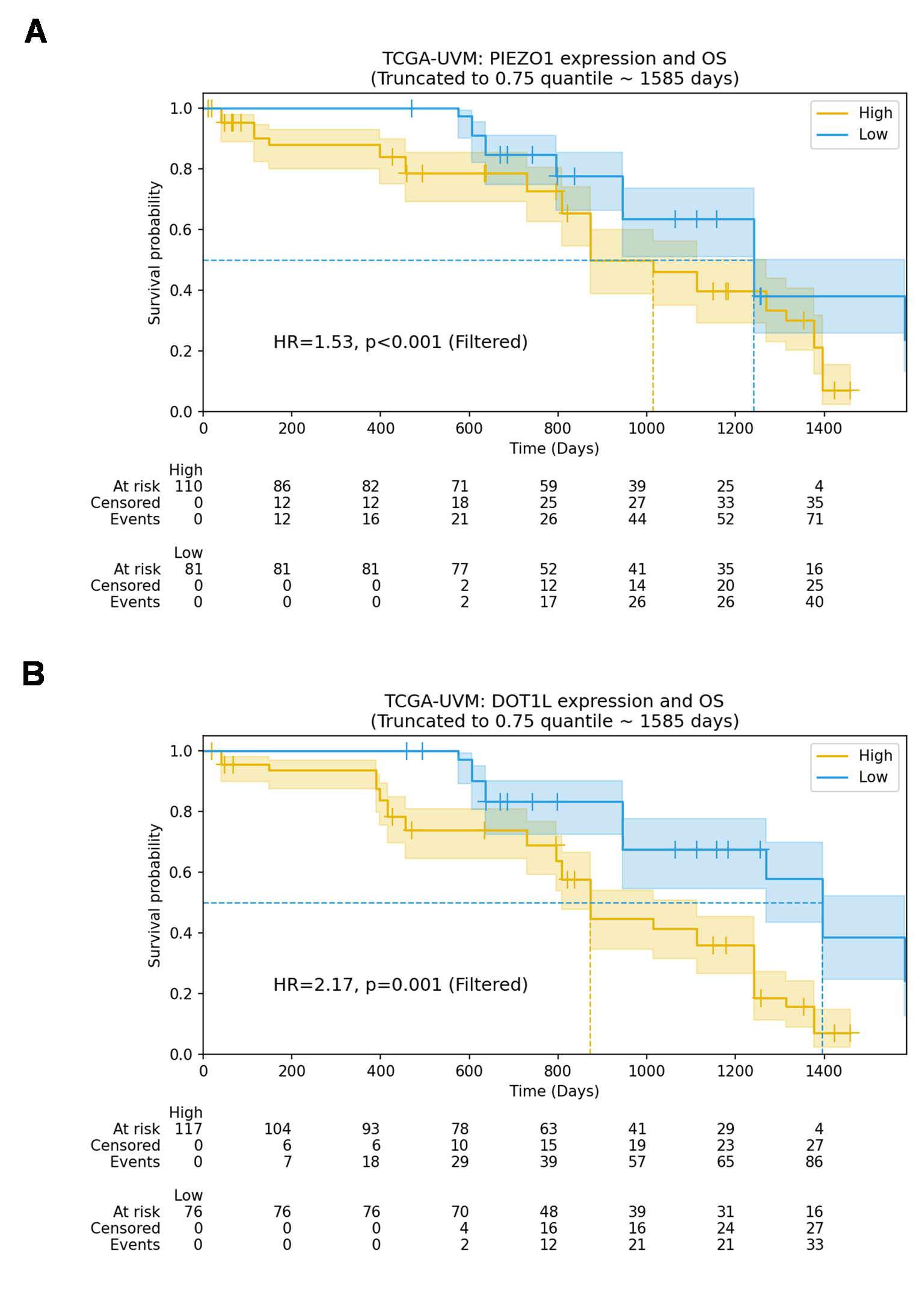


**Supplementary Figure S11.** Kaplan–Meier Survival Analysis. **(A)** High PIEZO1 expression correlated with poorer overall survival in the TCGA- UM cohort (HR = 1.53, *P* < 0.001). **(B)** High DOT1L expression correlated with poorer overall survival in the TCGA-UM cohort (HR = 2.17, *P* = 0.001).

**Supplementary tables**

**Supplementary Table S1.** Primers and shRNA sequences used in this study

| Primer | name Sequence (5’-3’) | Purpose |
| --- | --- | --- |
| *PIEZO1* | ATCCTGCAGAGGAACATGGC  TAGGACGTCTCCTTGTACCG | PCR |
| *DOT1L* | AGGAAGGTGGCAGAATCGTG  TGATGGTGCCGATGTCACTC | PCR |
| *SOX2* | AGTGGAAACTTTTGTCGGAGAC  GCAGCGTGTACTTATCCTTCTTC | PCR |
| *NANOG* | ACCAGAACTGTGTTCTCTTCCAC  AGGTCTTCACCTGTTTGTAGCTG | PCR |
| *GAPDH* | TGACTCTACCCACGGCAAGTTCAA  ACGACATACTCAGCACCAGCATCA | PCR |
| *DOT1L*-shRNA | CCTACAACGACCTGATTCA  TGAATCAGGTCGTTGTAGG | shRNA |
| *DOT1L*-OE | NM_032482.3 | OE |
| *PIEZO1*-shRNA | GCCGAGAGACAGAGAAGAAAT  CGGCTCTCTGTCTCTTCTTTA | shRNA |

**Supplementary Table S2.** Modulus of elasticity of PAAGs

| Acrylamide from 40% stock solution (mL) | Bisacrylamide from 2% stock solution (mL) | Water (mL) | Shear modulus,  G (kPa) | Young’s modulus,  E (kPa) |
| --- | --- | --- | --- | --- |
| 0.75 | 0.75 | 8.5 | 0.48 ± 0.03 | 1.43 ± 0.09 |
| 1.25 | 0.75 | 8 | 1.52 ± 0.08 | 4.56 ± 0.25 |
| 1.5 | 0.75 | 7.75 | 1.83 ± 0.04 | 5.49 ± 0.12 |
| 2 | 0.75 | 7.25 | 5.22 ± 0.52 | 15.67 ± 1.55 |
| 2.25 | 1 | 6.75 | 7.15 ± 0.33 | 21.46 ± 0.99 |
| 2.40 | 1 | 6.6 | 7.86 ± 0.13 | 23.58 ± 0.38 |
| 2.5 | 1.25 | 6.25 | 8.85 ± 0.07 | 26.54 ± 0.22 |
| 2.5 | 1.75 | 5.75 | 9.77 ± 0.23 | 29.32 ± 0.69 |
| 2 | 2.5 | 5.5 | 13.14 ± 0.41 | 39.43 ± 1.24 |

*Abbreviations: PAAGs: Polyacrylamide hydrogels

**Supplementary Table S3.** **Antibodies used in this study**

| **Antibody** | **Manufacturer** | **Category number** | **Uses** |
| --- | --- | --- | --- |
| PIEZO1 | Proteintech (Wuhan,China) | 28511-1-AP | WB/IF |
| NANOG | Proteintech (Wuhan,China) | 14295-1-AP | WB |
| NANOG | Thermo Fisher Scientific (Waltham, MA, USA) | PA1-097X | IF |
| SOX2 | Proteintech (Wuhan,China) | 66411-1-lg | WB |
| SOX2 | Abcam (Cambridge,MA,USA) | ab92494 | IF |
| DOT1L | Znbio (Chengdu,China) | R380920 | WB |
| DOT1L | Santa Cruz (CA, USA) | sc-390879 | IF |
| GAPDH | Proteintech (Wuhan,China) | 10494-1-AP | WB |
| Melan-A | Proteintech (Wuhan,China) | 18472-1-AP | IF |
